# ARMC5 controls the degradation of most Pol II subunits, and ARMC5 mutation increases neural tube defect risks in mice and humans

**DOI:** 10.1186/s13059-023-03147-w

**Published:** 2024-01-15

**Authors:** Hongyu Luo, Linjiang Lao, Kit Sing Au, Hope Northrup, Xiao He, Diane Forget, Marie-Soleil Gauthier, Benoit Coulombe, Isabelle Bourdeau, Wei Shi, Lucia Gagliardi, Maria Candida Barisson Villares Fragoso, Junzheng Peng, Jiangping Wu

**Affiliations:** 1 Centre de Recherche, Centre Hospitalier de l’Université de Montréal (CHUM), Montreal, QC Canada; 2 Department of Pediatrics, McGovern Medical School at the University of Texas Health Science Center at Houston (UTHealth) and Children’s Memorial Hermann Hospital, Houston, TX USA; 3 Department of Translational Proteomics, Institut de Recherches Cliniques de Montréal, Montreal, QC Canada; 4 Department of Biochemistry and Molecular Medicine, Université de Montréal, Montreal, QC Canada; 5 Division of Endocrinology, CHUM, Montreal, QC Canada; 6 Department of Medicine, Université de Montréal, Montreal, QC Canada; 7 Adelaide Medical School, University of Adelaide, Adelaide, Australia; 8 Endocrine and Metabolic Unit, Royal Adelaide Hospital, Adelaide, Australia; 9 Department of Genetics and Molecular Pathology, SA Pathology, Adelaide, Australia; 10 Endocrine and Diabetes Unit, Queen Elizabeth Hospital, Adelaide, Australia; 11 Unidade de Suprarrenal Disciplina de Endocrinologia E Metabologia, Hospital das Clínicas, Faculdade de Medicina da Universidade de São Paulo, São Paulo, Brazil; 12 Division of Nephrology, CHUM, Montreal, QC Canada

**Keywords:** ARMC5, Neural tube defects, Myelomeningocele, Ubiquitin ligase specific for all Pol II subunits, Pol II pool size, FOLH1

## Abstract

**Background:**

Neural tube defects (NTDs) are caused by genetic and environmental factors. ARMC5 is part of a novel ubiquitin ligase specific for POLR2A, the largest subunit of RNA polymerase II (Pol II).

**Results:**

We find that ARMC5 knockout mice have increased incidence of NTDs, such as spina bifida and exencephaly. Surprisingly, the absence of ARMC5 causes the accumulation of not only POLR2A but also most of the other 11 Pol II subunits, indicating that the degradation of the whole Pol II complex is compromised. The enlarged Pol II pool does not lead to generalized Pol II stalling or a generalized decrease in mRNA transcription. In neural progenitor cells, ARMC5 knockout only dysregulates 106 genes, some of which are known to be involved in neural tube development. FOLH1, critical in folate uptake and hence neural tube development, is downregulated in the knockout intestine. We also identify nine deleterious mutations in the ARMC5 gene in 511 patients with myelomeningocele, a severe form of spina bifida. These mutations impair the interaction between ARMC5 and Pol II and reduce Pol II ubiquitination.

**Conclusions:**

Mutations in ARMC5 increase the risk of NTDs in mice and humans. ARMC5 is part of an E3 controlling the degradation of all 12 subunits of Pol II under physiological conditions. The Pol II pool size might have effects on NTD pathogenesis, and some of the effects might be via the downregulation of FOLH1. Additional mechanistic work is needed to establish the causal effect of the findings on NTD pathogenesis.

**Supplementary Information:**

The online version contains supplementary material available at 10.1186/s13059-023-03147-w.

## Background

In this study, we report previously unknown relationships among three biological events, i.e., neural tube defects, the function of a molecule called ARMC5, and the degradation of DNA-directed RNA polymerase II (Pol II).

### Neural tube defects

During embryonic development, the neural plate folds into a neural tube to form the future brain and spinal column. The process occurs during days 17–28 of human gestation or e8.5–10.5 (embryonic days) in mice [1]. Defective closure of the neural tube causes various manifestations, such as *spina bifida*, anencephaly, encephalocele, and iniencephaly. The three latter forms of neural tube defects (NTDs) are very severe and frequently result in miscarriage or stillbirth. There are four principal types of *spina bifida*: meningocele, myelomeningocele (MM), myelocele, and spinal dysraphism. NTD is one of the most common birth defects, with median prevalence varying from 6.9 to 21.9 per 10,000 live-birth in different World Health Organization-designated regions [2]. During the process of neural tube closing, the neural plate cells need to undergo necessary proliferation, differentiation, morphological transformation, and migration [3–6]. These steps can be affected by genetic and environmental factors, which all contribute to NTD risks [7]. It has been well established that sufficient maternal dietary folate intake is essential for proper neural tube closing [8]. The genetic contribution to NTD is polygenic. Based on candidate gene studies, some genes in the folate metabolic pathways have been found to be associated with NTD risks in humans [9–11]. Mutations/deletions in more than 200 genes are known to cause NTD in mice [12], although only a limited number of them are associated with human NTD risks according to human genetic studies [13].

### ARMC5

ARMC5 is a protein containing an armadillo domain consisting of seven armadillo repeats. Each repeat is about 40 amino acids (aa) long and consists of three α-helices [14]. Human and mouse ARMC5 proteins share 90% aa sequence homology and have similar structures. Both have the ARMC domain towards their N-terminus and a BTB (Broad-complex, tramtrack, and bric-à-brac) domain towards their C-terminus [15–17]. The dominant human ARMC5 protein isoform is 935 aa in length (NP_001098717.1) [18], and mouse ARMC5, 926 aa (NP_666317.2). In humans, four other isoforms are derived from the same gene. Most isoforms vary in the 5′- and 3′- regions. One of the four isoforms has two extra exons at the 5′ end of the gene and translates into a longer protein isoform of 1030 aa in length (NP_001275696.1) [18]. ARMC5 does not have any enzymatic activities. It functions via its association with other proteins. We previously reported that ARMC5 was associated with a group of molecules, including Cullin-3 (CUL3) [19], according to a yeast 2-hybrid assay.

We recently reported the phenotype of *Armc5* gene knockout mice (KO mice) [19]. The KO mice were smaller from the fetal stage until old age and were born below the expected Mendelian ratio from heterozygous parents. The function of T lymphocytes of the KO mice was compromised in that they had reduced proliferation and differentiation in vitro, decreased autoimmune responses, and defective viral clearance in vivo. The KO mice presented adrenal gland hyperplasia in old age, similar to primary bilateral macronodular adrenal gland hyperplasia (PBMAH). Approximately 21–26% of PBMAH patients carry *ARMC5* mutations [20–23].

### Pol II

Pol II is responsible for transcribing all the mRNA and most small nuclear RNA, microRNA, and long non-coding RNA [24, 25]. Pol II is highly conserved. Human and mouse Pol II both have 12 subunits [26]. POLR2A is the largest catalytic subunit. Pol II might pause during mRNA transcription for various reasons, such as template DNA damage, cell stress, or gene activation status. Once these adverse conditions are resolved, it will continue its journey along the template DNA. Permanent Pol II stalling will block the transcription, and the stalled Pol II needs to be removed to resume transcription. It is believed that ubiquitination, followed by proteasome degradation, is a process to remove the stalled Pol II [27–29]. It follows that if such permanently stalled Pol IIs are not degraded, there will be a generalized transcription depression.

How the Pol II pool size homeostasis is maintained and whether an abnormal Pol II pool size plays a pathogenic role are an understudied area. A related critical question is whether an abnormal Pol II pool size affects all the genes or just a subset of genes. Recently, Vidakovic et al. and Nakazawa et al. reported that K1268 ubiquitination is necessary and sufficient for POLR2A degradation in cells after UV irradiation. K1268R mutation prevents POLR2A ubiquitination, resulting in POLR2A accumulation and an enlarged Pol II pool size in cells with irradiation-induced massive DNA damage [30, 31]. The enlarged Pol II pool in the mutant cells in this model selectively leads to faster transcription recovery of short genes and upregulates a subset of genes (about 1600 genes) [30]. These studies demonstrate that the effect of Pol II pool size is not universal to all the genes in the irradiated cells. However, we do not know whether the same is true under a physiological condition without massive DNA damage.

### Pol II degradation 

Ubiquitination is involved in protein degradation and function. Protein ubiquitination is catalyzed by a cascade of enzymes, i.e., E1 (Ub-activating enzyme), E2 (Ub-conjugating enzyme), and E3 (Ub ligase) [32]. The specificity of the cascade is determined by E3, which has three families: Ring-finger (single or multiple subunits), HECT, and RBR [33]. The Ring-finger E3s are the largest family. A numerous subunit Ring-finger E3 contains a RING-finger protein (e.g., ROC1 or RBX1), a Cullin (CUL) protein (CUL1, 2, 3, 4A, 4B, 5, and 7), and a substrate recognition unit [34].

Due to the central role of Pol II and its largest subunit, POLR2A, in cell biology, POLR2A-specific E3 is of vital interest to cell biologists. Several such E3s have been reported before, but most of them only have convincing activities in cultured cells after irradiation- or drug-induced massive DNA damage [35–40]. Two of these E3s do have activity in unmanipulated cell lines, but such observation has not extended to tissues and organs [38, 41]. None of these POLR2A-specific E3s have known effects on the degradation of the other 11 Pol II subunits, either after massive DNA damage or under physiological conditions.

It is to be noted that in most of the previous studies related to Pol II function and pool size, POLR2A has been used as a surrogate marker of Pol II, and the levels of the other 11 subunits are rarely assessed. To our knowledge, only three prior publications have addressed the ubiquitination and degradation of other Pol II subunits. An E3 Asr1 in yeasts can mono-ubiquitinate POLR2B in vitro, probably via its interaction with POLR2A [42], although whether this E3 affects the POLR2B protein level in vivo is not known. BRCA1 is reported to ubiquitinate POLR2H after massive DNA damage, but it does not affect the protein level of POLR2H in whole-cell lysates [43]. VHL-containing E3 ubiquitinates POLR2G and controls its degradation in the absence of massive DNA damage [44].

In the present work, we revealed that a novel ARMC5-containing E3 was essential for the degradation of most of the 12 subunits of Pol II and thus controlled the homeostasis of the whole Pol II complex under physiological conditions. Failed Pol II degradation due to ARMC5 deletion did not result in generalized Pol II stalling nor generalized transcription depression. The abnormally large Pol II pool in the KO neural precursor cells and intestine dysregulated the transcription of a subset of genes, some of which are known to be critical in neural tube development. A human genetic study discovered nine highly deleterious single-nucleotide variants (SNVs) in the ARMC5 exons of myelomeningocele (MM) patients; four of the nine SNVs were proven essential for the POLR2A-specific E3 activity.

## Results

### Increased incidence of NTD in Armc5 KO mice

**Fig. 1:**
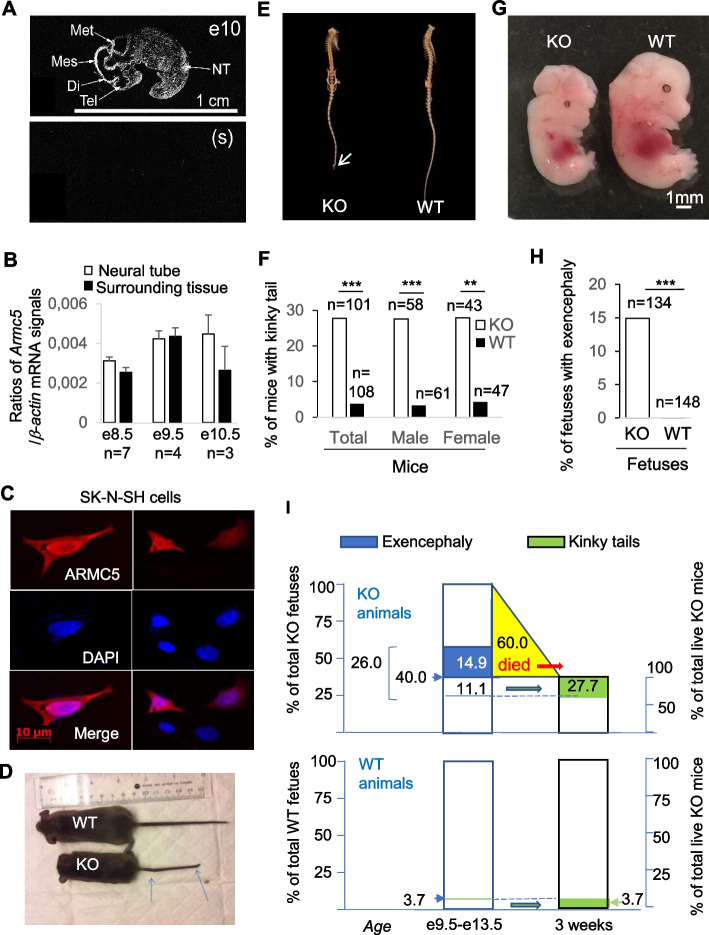
Increased incidence of NTD in Armc5 KO mice. **A** *Armc5* expression in the neural tube of an e10 WT C57BL/6 fetus according to X-ray autoradiography of in situ hybridization. NT: rostral neural tube; Met: metencephalon; Mes: mesencephalon; Di: diencephalon; Tel: telencephalon. S: sense. **B** *Armc5* expression in the e8.5, e9.5, and e10.5 neural tubes and adjacent tissues in WT embryos according to RT-qPCR. The means ± SD of signal ratios of *Armc5* versus β*-actin* based on 4 independent biological samples are shown. **C** ARMC5 was detected in both cytosol and nuclei. *ARMC5*-HA-expressing plasmid-transfected SK-N-SH neuronal cells were fixed 48 h after transfection. The presence of ARMC5 in the nuclei and cytosol was determined by immunofluorescence. Representative micrographs from three independent experiments are shown. ARMC5-HA: pseudo-red; nucleus staining by DAPI: pseudo-blue. **D** A KO mouse with a kinky tail (arrows: kinks in the tail). **E** Skeletons of WT and KO mice according to micro-CT scan. Arrow: the curled tail in a KO mouse. **F** Increased incidence of KO mice with kinky tails on the day of weaning (3 weeks old). The numbers of male, female, and total mice examined are indicated. **G** An e12.5 KO fetus with exencephaly. **H** Increased incidence of exencephaly in e9.5 to e13.5 KO fetuses. The number of KO and WT fetuses examined on e9.5 to e13.5 is indicated. **I** A summary of the incidence of NTD in e9.5 − e13.5 fetuses and 3-week-old WT and KO animals. The percentages of NTD among total e9.5 − e13.5 KO and WT fetuses and the percentages of NTD among 3-week-old KO and WT pups are shown. E9.5–13.5 fetuses with exencephaly are in blue, and live pups with kinky tails at 3 weeks are in green. Fetuses/mice in yellow are those who died between e9.5 and e13.5 and weaning at 3 weeks. **: *p* < 0.01; ***: *p* < 0.001 (*χ*^2^ test)

To explore unknown functions of a molecule, we often first assess its expression location. In situ hybridization revealed that *Armc5* had generalized expression in all the tissues in the e10 embryo and highly expressed in the mouse e10 neural tube (Fig. 1A). RT-qPCR showed that *Armc5* was expressed in the neural tubes as well as their surrounding tissues on e8.5 and e9.5 (Fig. 1B). On e10.5, according to RT-qPCR, *Armc5* expression in the whole neural tube tended to be higher than that in the surrounding tissues, but the difference did not reach a significant level at the present sample size (*n* = 3) (Fig. 1B). This is compatible with the in situ finding that on e10, only in a small section of the neural tube *Armc5* expression was higher than the surrounding tissues.

We previously reported that ARMC5 was expressed predominantly in the cytosol [19]. However, we later found that if nuclear export was blocked, ARMC5 appeared in the nuclei of transfected HEK293 cells [45]. To determine the location of ARMC5 expression in neuronal cells, we transfected SK-N-SH neuronal cells with ARMC5-expressing constructs. Tagged ARMC5 was found in both cytosol and nuclei (Fig. 1C). The microscopy image was deposited in Figshare [46].

The presence of ARMC5 in the neural tube of embryos and in the nuclei of cells prompted us to assess the role of *Armc5* in neural tube development and its activity in the nuclei. In addition to smaller body sizes, as reported before [19], live-born *Armc5* KO mice in the CD1 x C57BL/6 F1 background presented significantly high incidences of kinky tails, a form of NTD [47, 48], upon visual inspection (Fig. 1D) or micro-CT imaging (Fig. 1E). Kinky tails were observed in 27.7% of KO mice at weaning, compared to 3.7% of the wild-type (WT) counterparts (Fig. 1F). The incidence rates did not show any apparent sex bias, with 27.6 and 27.9% in male and female KO mice, respectively.

Between e9.5 and e13.5, about 14.9% of KO embryos but 0% of WT embryos manifested exencephaly, a severe form of NTD. A representative image of an e12.5 KO fetus with exencephaly is shown in Fig. 1G, and a bar graph summarizing the incidence of KO and WT fetuses between e9.5 and e13.5 is presented in Fig. 1H.

The KO mice from heterozygous male and female parents were born below the Mendelian ratio. Only about 10% of the live pups at weaning were KO instead of the expected 25%. This suggests a high degree (15/25 = 60%) of embryonic/perinatal lethality in KO mice. At e13.5, the percentage of KO fetuses (including those with exencephaly) was still at the expected Mendelian ratio of 25%. Thus, 60% of KO fetuses/newborns must have died between e9.5 and 13.5 and weaning (Fig. 1I; yellow triangle). The mice with kinky tails at weaning (27.7%) represented 11.1% of the KO fetuses at e9.5–e13.5 (Fig. 1I, upper panel). Hence, the NTD incidence (14.6% with exencephaly, which must have died before birth, plus 11.1% live mice with kinky tails) is 26% among all the KO mice (dead or live) (Fig. 1I, upper panel), compared to 3.7% among the WT mice (Fig. 1I, lower panel).

Heterozygous embryos/fetuses and mice had no increased NTD incidence compared to the WT ones.

The results of this section indicate that ARMC5 mutation is an NTD risk modifier in mice.

### Decreased proliferation and increased apoptosis of KO cells implicated in neural tube development

**Fig. 2:**
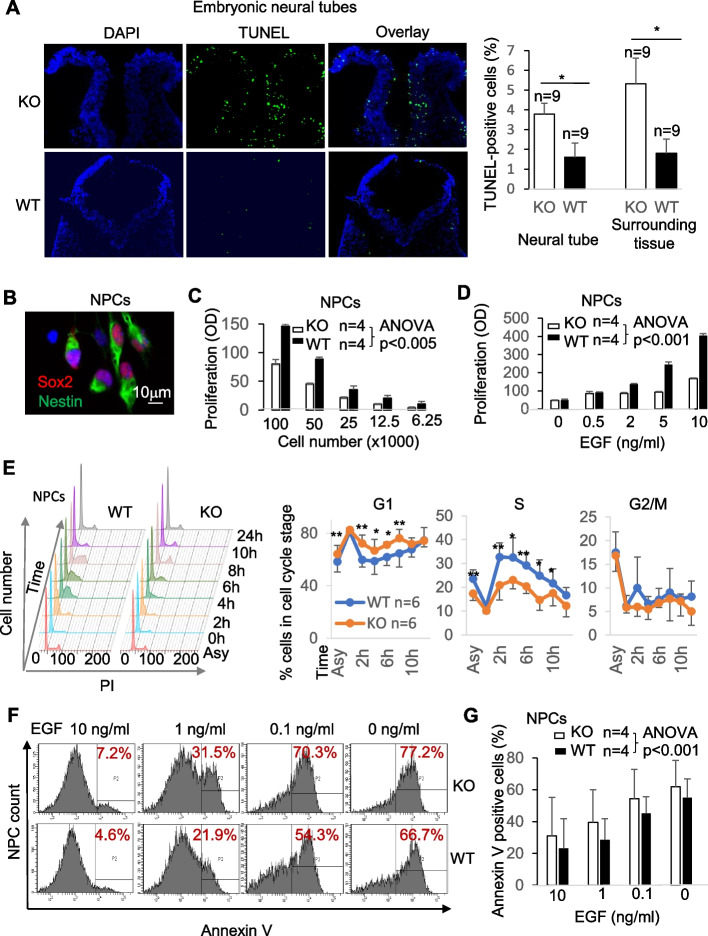
The increased apoptosis and reduced proliferation of cells in KO neural tubes and NPCs. **A** Increased cell apoptosis in KO neural tubes. Neural tubes at the level of the rostral hindbrain (at upper 1/3 of the body length measured from the cranial extreme; sectioned transversally) of e9.5 KO embryos with exencephaly and normal WT counterparts were determined by TUNEL. The percentage (means ± SD) of TUNEL-positive cells among total cells in randomly selected view areas in the neural folds and adjacent areas is presented in a bar graph at the right. Results were based on the counting of three different sections per fetus (KO: 3 fetuses; WT: 3 fetuses). **B** Characterization of NPCs. WT NPCs were stained with NPC markers Sox2 (pseudo-red) and Nestin (pseudo-green). **C, D** Reduced KO NPC proliferation. Different numbers of NPCs, as indicated, were cultured in the presence of a fixed concentration of EGF (20 ng/ml) (**C**), or a fixed number of NPCs were cultured in the presence of different concentrations of EGF as indicated (**D**). After 72 h, proliferation was measured by an MTS-based CellTiter96 AQueous Assay. Samples were in triplicate, and means ± SD of OD_490 nm_ (after subtracting background absorbance based on OD_490 nm_ of wells without cells) of a representative experiment out of four independent ones are shown. **E** Slower progression of KO NPCs from the G1 phase to the S phase. NPCs were blocked at the late G1 phase with aphidicolin for 8 h. The cells were then released from the blockages, and their DNA content was measured by flow cytometry at different time points as indicated. A set of representative histograms is shown at the left, with DNA content stained with PI (propidium iodine). Results from 6 independent experiments are summarized at the right, with mean ± SD of the percentage of cells in the G1, S, and G2/M phase at different time points indicated. **F** Augmented apoptosis of KO NPCs cultured in the presence of different concentrations of EGF. NPCs were cultured for 20 h, and their apoptosis was measured by annexin V staining followed by flow cytometry. Representative histograms are shown (left panel). **G** A summary of the flow cytometry data (means ± SD) of four independent experiments is presented in a bar graph (right panel). Data in **A** and **E** were analyzed with paired two-way Student’s *t* test. *p < 0.05; **p < 0.01. Data in **C**, **D**, and **G** were analyzed by two-factor with replication ANOVA, and *p*-values for the WT versus KO groups were indicated

Neural tube closing requires the coordination of many cellular processes, such as proliferation and apoptosis. We thus evaluated these processes in the KO cells that were implicated in neural tube development. The apoptosis of cells in the e9.5 neural plate was evaluated using fluorescent terminal deoxynucleotidyl transferase dUTP nick end-labeling (TUNEL) assay (Fig. 2A). The microscopy image was deposited in Figshare [49]. The number of TUNEL-positive apoptotic cells (pseudo-green) in the neural plate at the rostral hindbrain level, as well as those in the surrounding tissues of the KO mice with exencephaly, was significantly higher than that of the WT counterparts. It is to be noted that only KO embryos with exencephaly were assessed for apoptosis in their neural plates. This was because we only wanted to test KO embryos that had NTD. Only those with exencephaly were the ones who surely had NTD. At e9.5, the kinky tail phenotype had not appeared yet, and most (74%) of the KO embryos did not have NTD. Randomly testing KO embryos would include many that had no NTD.

It is very challenging to obtain neural tube cells in sufficient numbers and homogeneity for biochemical analysis. An alternative to conducting biochemical studies of neural tube development at the molecular level is to use neural stem cells and neural progenitor cells (collectively called neural progenitor cells (NPCs) in this work), which are known to be involved in neural tube development [50, 51]. We isolated these cells from the e13.5 KO and WT brains and spinal cords and expanded them in vitro for 10–12 days according to the standard NPC preparation protocol [52]. The WT and KO e13.5 brains used for NPC preparation were harvested based on genotype. As long as the NPCs keep the progenitor cell characteristics, they resemble the NPCs presented in the neural tubes. SOX2 and NESTIN are markers of NPCs, and their expression in the KO and WT NPCs was similar according to RNA-seq (GEO; accession number GSE169350)[53]. The purity of WT and KO NPCs we prepared was routinely about 85%, according to SOX2 and NESTIN staining. A typical SOX2 and NESTIN staining of WT NPCs is presented in Fig. 2B. The microscopy image was deposited in Figshare [54]. These NPCs were used to study their proliferation and apoptosis in this section, as well as their transcriptome and POLR2A ChIP-seq. NPC growth is EGF (epidermal growth factor)-dependent in vivo and in vitro [55, 56]. The KO NPCs proliferated significantly slower than WT ones at different input cell numbers in the presence of EGF (Fig. 2C). To test the responsiveness of WT and KO NSCs to EGF stimulation, we also cultured the NSCs at a constant input cell number but at different EGF concentrations. KO NSCs responded poorly to EGF (Fig. 2D). To further prove the proliferative defect of KO NPCs, we showed that KO NPCs synchronized at the G1 phase progressed slower into the S phase, compared to their WT counterparts (Fig. 2E). At all the time points after the release, except 0 and 24 h, fewer KO cells were found in the S phase, indicating slower S phase entry. Conversely, more G1 phase KO NPCs were found at most of these time points, as expected.

NPCs will undergo apoptosis upon sudden EGF withdrawal [57]. This is a convenient model to assess NPC apoptosis. EGF withdrawal-induced apoptosis of KO and WT NPCs was measured by annexin V staining followed by flow cytometry. Representative histograms are shown (Fig. 2F), and a bar graph quantifies the results of all the independent experiments (Fig. 2G). Both KO and WT NPCs manifested an increased apoptosis rate inversely correlated to EGF concentrations, but KO NPCs showed a significantly higher degree of apoptosis.

These results indicate that ARMC5 deletion compromises the proliferation and induces apoptosis of cells involved in neural tube development in mice.

### ARMC5 physically interacts with CUL3 and POLR2A

**Fig. 3:**
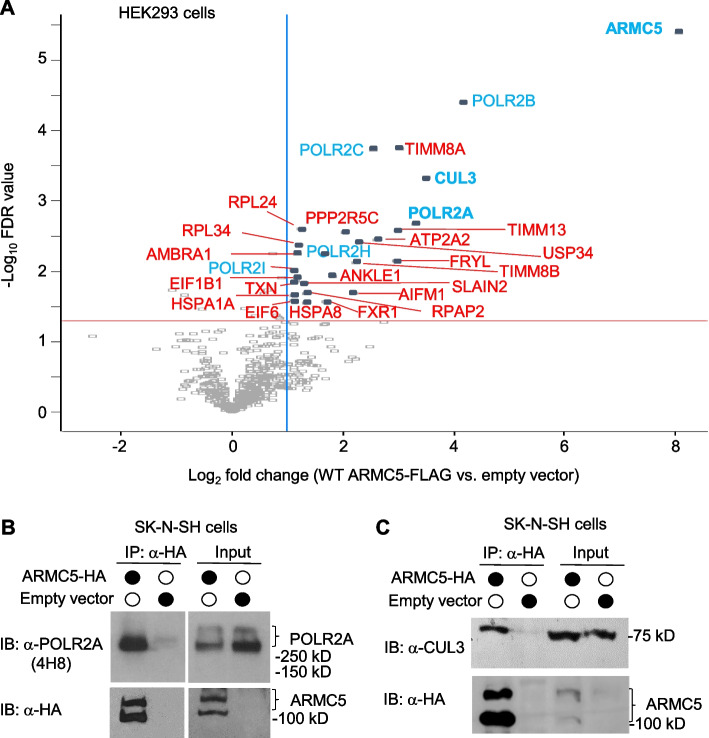
ARMC5 interacted with CUL3 and POLR2A. **A** A volcano plot showing ARMC5 interacted with endogenous CUL3 and POLR2A in HEK293 cells. HEK293 cells were transfected with ARMC5-FLAG-expressing plasmids. The anti-FLAG precipitates were analyzed by LC-MS/MS. The vertical line indicates twofold changes, and the horizontal line, FDR = 0.05, is based on three biological replicates. **B,C** ARMC5 interacted with endogenous POLR2A and CUL3 in SK-N-SH neuronal cells. SK-N-SH cells were transfected with a human ARMC5-HA-expressing plasmid or an empty vector. The presence of endogenous POLR2A (**B**) and CUL3 (**C**) in the immunoprecipitates was revealed by immunoblotting (IB) with anti-POLR2A and anti-CUL3 Abs. The presence of ARMC5-HA in the cell lysates and immunoprecipitates was confirmed by IB with anti-HA Ab (lower rows of **B** and **C**). The experiments were conducted more than three times, and representative results are shown

We previously conducted a yeast 2-hybrid assay to identify ARMC5-binding proteins. Seventeen significant hits were obtained. CUL3 and POLR2A were among the top six on the list [19]. To validate these findings, we transfected HEK293 cells with FLAG-tagged ARMC5-expressing plasmids. CUL3 and POLR2A were significantly associated with ARMC5 (false discovery rate (FDR) < 0.05 and fold change > 2) according to anti-FLAG Ab immunoprecipitation followed by liquid chromatography with tandem mass spectrometry (LC–MS/MS) (Fig. 3A). The dataset is available in proteomeXchange (accession number PXD047533) [58]. This was consistent with our previous immunoprecipitation/immunoblotting results in HEK293 cells [45]. However, the current LC–MS/MS results revealed a new finding that multiple other Pol II subunits (i.e., POLR2B, 2C, 2H, and 2I) in addition to POLR2A were also associated with ARMC5, suggesting a possibility that these Pol II subunits are also substrates of this novel E3.

Additional validation of the interaction among ARMC5, CUL3, and POLR2A was carried out employing immune precipitation followed by immunoblotting in neuronal cells, which were more relevant to NTD than HEK293 cells. SK-N-SH human neuronal cells were transfected with plasmids expressing human ARMC5-HA. The cell lysates were precipitated with anti-HA Ab and then immunoblotted with anti-POLR2A Ab (Fig. 3b) or anti-CUL3 (Fig. 3C). Endogenous POLR2A (Fig. 3B) and CUL3 (Fig. 3C) were detected in the precipitates, confirming that the ARMC5 physically interacted with these two molecules. In the ARMC5 immunoblotting, there were always two prominent bands, one at 130 kD and the other at 100 kD. The smaller band’s intensity varied in different experiments. Our previous study using step-wise deletion of ARMC5 confirmed that the 100-kD fragment was a cleavage product of the full-length larger 130-kD ARMC5 but not an isoform initiated from a downstream start codon during translation [45].

The results of this section show that ARMC5 interacts with both CUL3 and POLR2A.

### ARMC5 was part of POLR2A-specific E3 responsible for POLR2A degradation under a physiological condition

CUL3 is often part of a multiple-subunit RING-finger E3 complex, in which CUL3 interacts with a RING-finger protein RBX1 [59]. In such complexes, CUL3 also interacts with a BTB domain-containing protein, which serves as an E3 substrate recognition subunit [60]. Since ARMC5 contained a BTB domain towards its C-terminus and interacted with CUL3 and POLR2A, we hypothesized that it was the substrate recognition subunit of an E3 whose substrate was POLR2A.

**Fig. 4:**
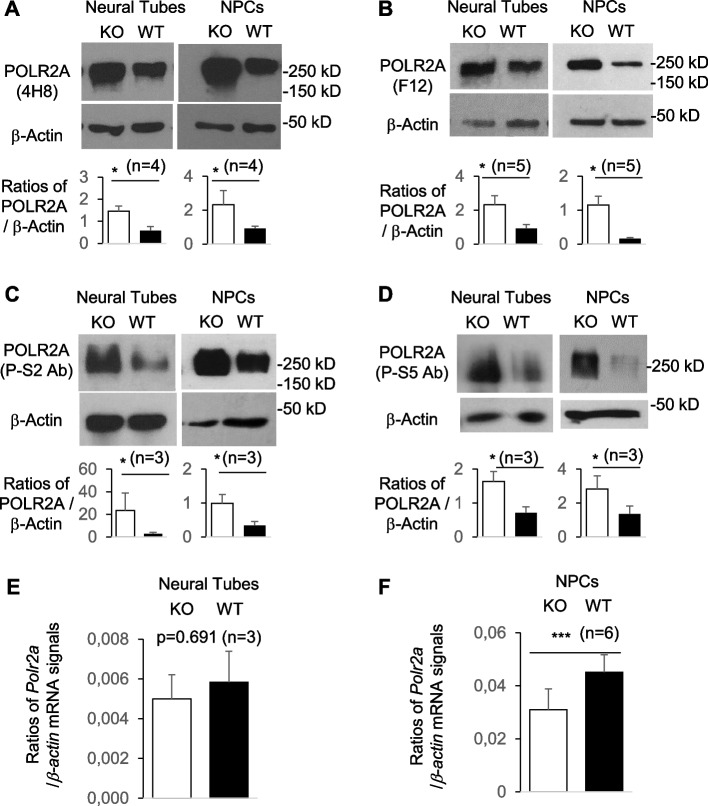
POLR2A protein accumulation in KO neural tubes and NPCs. **A, B** Increased levels of hyper- and hypo-phosphorylated POLR2A protein (**A**; recognized by mAb 4H8) and total POLR2A protein (**B**; recognized by anti-N-terminal mAb F12) in e9.5 KO neural tubes and KO NPCs according to immunoblotting. **C,D** Increased levels of S2-phosphorylated POLR2A (**C**; recognized by mAb E1Z3G) and S5-phosphorylated POLR2A (**D**; recognized by mAb D9N5I) in e9.5 KO neural tubes and KO NPCs. Representative immunoblots are shown. Bar graphs (mean ± SD) in the lower panels from **A** to **D** summarize the results of 3–5 independent experiments for each of these panels. **E** *Polr2a* mRNA levels of KO and WT e9.5 neural tubes were similar according to RT-qPCR. **F** *Polr2a* mRNA levels in the KO NPCs were lower than the WT counterparts. In **E** and **F**, the ratios (means ± SD) of signals of the testing molecules versus that of β*-actin* are shown. Data were analyzed by paired two-way Student’s *t* tests. *p < 0.05; ***p < 0.001

One of the consequences of protein ubiquitination, particularly K48-linked ubiquitination, is to channel substrate proteins to the proteasome for degradation [32]. If ARMC5-CUL3-RBX1 is a POLR2A-specific E3, we might observe an accumulation of POLR2A protein in the ARMC5 KO tissues. The C-terminal domain (CTD) of POLR2A of humans and mice contains 52 tandem heptapeptide repeats (Tyr1-Ser2-wujianpPro3-Thr4-Ser5-Pro6-Ser7). The phosphorylation or the lack of it in different serine residues at the CTD reflects Pol IIs at different stages of transcription. POLR2A without CTD phosphorylation is present in Pol II at the preinitiation stage at the promoter. S5 phosphorylation occurs when POLR2A is at the beginning of the transcription, i.e., at the transcription start site (TSS), while S2 phosphorylation occurs when Pol II moves towards the end of the gene, i.e., at the transcription end site (TES) [61]. MAb F12 binds to the N-terminus of POLR2A regardless of the latter’s phosphorylation status, and such PLOR2A is in all the Pol IIs that are on the whole length of genes, i.e., at the promoter region, TSS, gene body, and TES. MAb 4H8 recognizes POLR2A with hyper and hypo CTD phosphorylation. This mAb is slightly different from mAb F12 in that the former does not bind to the un-phosphorylated POLR2A at the preinitiation stag. Our immunoblotting results showed that hyper- and hypo-phosphorylated POLR2A (recognized by mAb 4H8) (Fig. 4A), the total POLR2A (recognized by mAb F12) (Fig. 4B), POLR2A with CTD S2 phosphorylation (recognized by anti-P-S2 mAb) (Fig. 4C), and POLR2A with CTD S5 phosphorylation (recognized by anti-P-S5 mAb) (Fig. 4D) in KO neural tubes and NPCs were all increased. These results indicate that POLR2As at different stages of transcription are all increased under a physiological condition. The mRNA levels of POLR2A in the KO neural tubes (Fig. 4E) and NPCs (Fig. 4F) were not upregulated. This confirms that the increased POLR2A protein levels were a post-transcription event. As a matter of fact, the POLR2A mRNA level in the KO NPCs were even reduced (Fig. 4F) due to some unknown mechanisms after ARMC5 deletion. As a reduced mRNA level normally translates into a reduced protein level, the increased POLR2A protein level in the KO NPCs, accompanied by their reduced POLR2A mRNA level, suggests that the post-translational upregulation of POLR2A is more prominent than it appears to be.

**Fig. 5:**
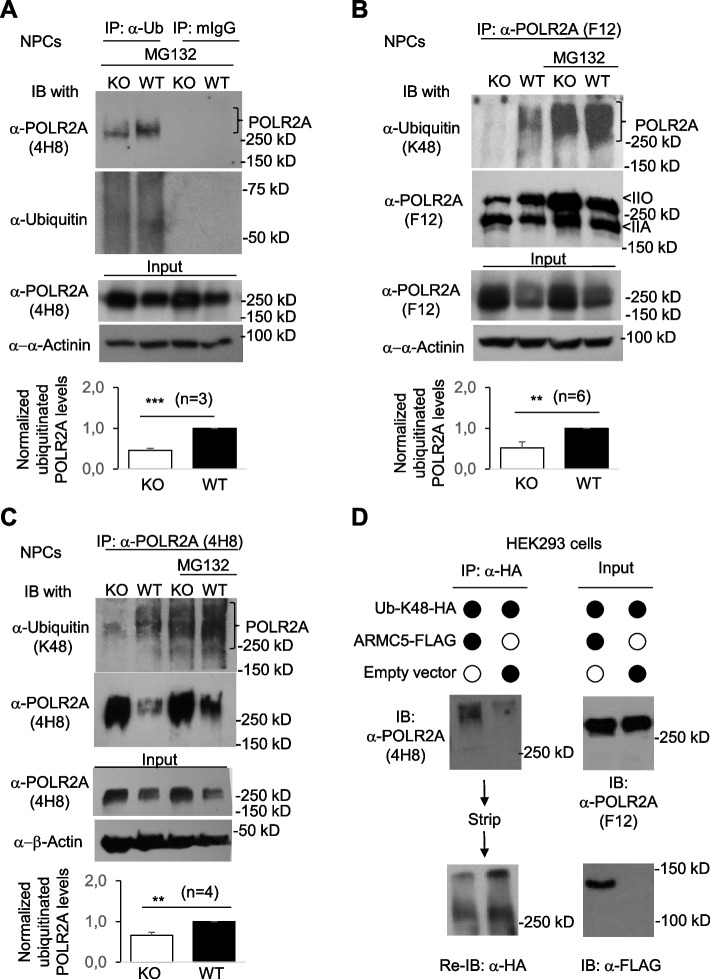
POLR2A-CUL3-RBX1 as a novel POLR2A-specific E3. **A–C** Reduced POLR2A ubiquitination in KO NPCs. KO and WT NPCs were cultured in the absence or presence of MG132 (10 µM). **A** All the ubiquitinated proteins were precipitated by anti-ubiquitin Ab or mouse IgG isotype control and then blotted with anti-POLR2A mAb 4H8 or anti-ubiquitin antibody. **B**,**C** The total POLR2A or POLR2A with high and low CTD phosphorylation was precipitated with mAb F12 (**B**) or mAb 4H8 (**C**), respectively. Their K48-linked ubiquitination was determined by blotting with anti-K48-linked ubiquitin Ab. **D** ARMC5 overexpression augmented K48-linked POLR2A ubiquitination. HEK293 cells were transfected with plasmids expressing FLAG-tagged ARMC5 and HA-tagged mutant ubiquitin only capable of K48-linked polyubiquitination. Proteins with K48-linked ubiquitination were precipitated with anti-HA Ab, and K48-linked POLR2A in the precipitates was detected by anti-POLR2A mAb (4H8) (upper left panel). Empty vectors were used as controls in transfection. The membrane was stripped and reblotted for total K48-linked proteins as loading control of the precipitated proteins (lower left panel). The total POLR2A and FLAG-ARMC5 expression in the lysates was detected by anti-total POLR2A mAb (F12) and anti-FLAG Ab, respectively (right panels). All experiments were repeated three times, and representative ones are shown. The bar graphs (means ± SD) in **A–C** summarize the results of three or more independent experiments for each panel. IP: immunoprecipitation; IB: immunoblotting. **p < 0.01; ***p < 0.001 (two-way Student’s *t* tests)

Is POLR2A accumulation in the KO cells due to compromised ubiquitination? We evaluated POLR2A ubiquitination in KO NPCs. Total POLR2A ubiquitination was reduced in KO NPCs in the presence of proteasome inhibitor MG132, which prevented rapid degradation of ubiquitinated proteins by the proteasome), supporting our hypothesis that ARMC5 is part of a POLR2A-specific E3 (Fig. 5A). K48-linked ubiquitination is the major type of polyubiquitination for proteasome-mediated protein degradation [62]. K48-linked POLR2A ubiquitination was thus evaluated. Total POLR2A in the KO NPCs was precipitated by F12 mAb, which binds POLR2A regardless of its CTD phosphorylation status and then blotted with Ab against K48-linked ubiquitin. In this experiment, a limited amount of F12 mAb was used to pull down a similar amount of POLR2A in WT and KO NPC samples (second row, Fig. 5B). The results showed that the KO NPCs had significantly lower levels of K48-linked ubiquitination of total POLR2A (top row, Fig. 5B). A similar result was obtained when mAb 4H8, which is against both hyper- and hypo-phosphorylated POLR2A, was used for immunoprecipitation (Fig. 5C). In these experiments (Fig. 5BC, ), the presence of proteasome inhibitor MG132 augmented the signals of ubiquitinated POLR2A, indicating that the proteasome constantly removed K48-linked POLR2A in the absence of the inhibitor. The presence of K48-linked ubiquitinated POLR2A in the KO cells is probably caused by some other E3s.

Using a different approach to prove the role of ARMC5 on POLR2A ubiquitination, we overexpressed FLAG-tagged ARMC5 in HEK293 cells, along with an HA-tagged mutant ubiquitin that only allowed K48-linked polyubiquitination. ARMC5 overexpression in these cells resulted in enhanced K48-linked POLR2A ubiquitination (Fig. 5D). This result conversely corroborates the conclusion obtained from KO NPCs.

The results in this section reveal that ARMC5 physically interacts with CUL3 and POLR2A and is part of a POLR2A-specific E3 under a physiological condition. This E3 controls POLR2A degradation by the latter’s K48-linked ubiquitination and shows no discrimination against the POLR2A CTD phosphorylation status.

### ARMC5 controls the degradation of most subunits of Pol II under physiological conditions

**Fig. 6:**
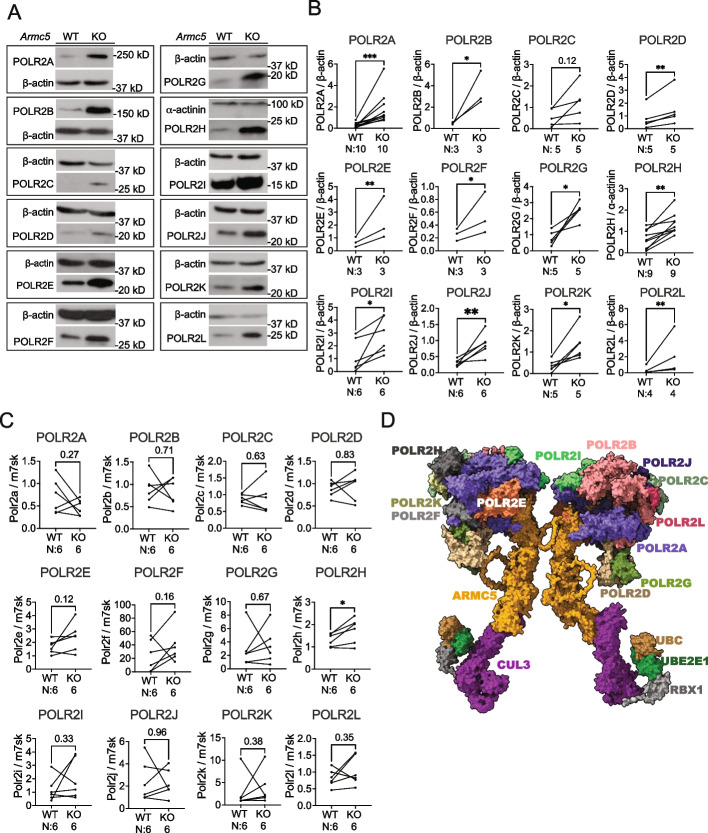
ARMC5 controls the degradation of most Pol II subunits under physiological conditions. **A**,** B** ARMC5 KO resulted in the accumulation of the 12 Pol II subunits. The protein levels of all 12 Pol II subunits (POLR2A-2L) in WT and KO MEF cells were determined by immunoblotting. Representative immunoblotting is shown in **A**. Pol II subunits, β-actin and α-actinin signals were quantified by densitometry, and signal ratios of the subunits versus β-actin or α-actinin of paired WT and KO samples (linked by lines) are presented in **B**. **C** mRNA levels of most Pol II subunits remained unchanged in KO MEFs. The mRNA levels of the subunits in the WT and KO MEFs were measured by RT-PCR, and the results are shown as the signal ratios of subunits versus *Rn7sk*. The number (*n*) of independent experiments is indicated. *p < 0.05; **p < 0.01; ***p < 0.001 (two-way Student’s *t* tests). **D** A 3D model of the Pol II and ARMC5-CUL3-RBX1 E3 complex. This dimeric E3 is shown to interact with two Pol IIs via POLR2A. The protein structures of all the components were obtained from PDB and AlphaFold2. The components are positioned in this 3D model according to the existing structural information from PDB and our previous binding and deletion studies [45]. Only the visible Pol II subunits in this viewing angle are marked

The presence of multiple Pol subunits in the ARMC5 precipitates according to proteomics (Fig. 3A) raised an intriguing probability that this novel ARMC5-containing E3 was involved in the degradation of not only POLR2A but also other Pol II subunits that are associated with POLR2A. We assessed the protein levels of all 12 Pol II subunits in the WT and KO mouse embryonic fibroblasts (MEFs). Quite unexpectedly, in the KO MEFs, all the subunits were drastically accumulated according to immunoblotting (Fig. 6A). The statistical analyses are presented in Fig. 6B. Although POLR2C had not reached statistical significance, probably due to a high degree of inter-experimental variation, the tendency of the increase in the KO cells is obvious. The mRNA levels of these subunits in the KO and WT MEFs had no significant difference except POLR2H, which had a very moderate increase in the KO MEFs (Fig. 6C). These results indicate compromised degradation of most, if not all, the Pol II subunits, in the absence of ARMC5.

Based on our novel results presented in this study, our previous detailed deletion studies of interacting regions among ARMC5, POLR2A, and CUL3 [63], and the structural information found in the Protein Data Bank and AlphaFold2 [64, 65], we constructed a 3D model for a complex containing this multi-subunit RING Finger family E3 and its substrate Pol II (Fig. 6D). In this complex, ARMC5 functions as the substrate (POLR2A) recognition subunit of the E3, which likely acts not only on its direct target POLR2A but all the other Pol II subunits in its vicinity. Our previous study demonstrated that this E3 forms a dimer, with two ARMC5s linked together via their ARM domains [45]. Thus, a dimeric ARMC5-containing E3 interacting with two Pol II complexes is illustrated in this 3D model.

### The impact of ARMC5 KO on the NPC transcriptome

The significant accumulation of almost all the Pol II subunits in the KO cells suggests an enlarged Pol II pool size. Since most Pol IIs in the nuclei are known to engage with the genes [66], this enlarged Pol II pool also likely does so.

The consequence of an enlarged Pol II pool is a topic that is not well studied. Is there generally decreased transcription due to the failure to degrade stalled Pol II or a generally increased transcription because more Pol IIs are available? To answer these questions, we conducted RNA sequencing (RNA-seq) of KO and WT NPCs to evaluate their transcriptome. Forty-seven thousand fifty-nine transcripts from 16,475 genes showed detectable expression in NPCs after filtering out those with less than one count per million reads. The RNA-seq dataset is available in the Gene Expression Omnibus (GEO; accession number GSE169350) [53].

**Fig. 7:**
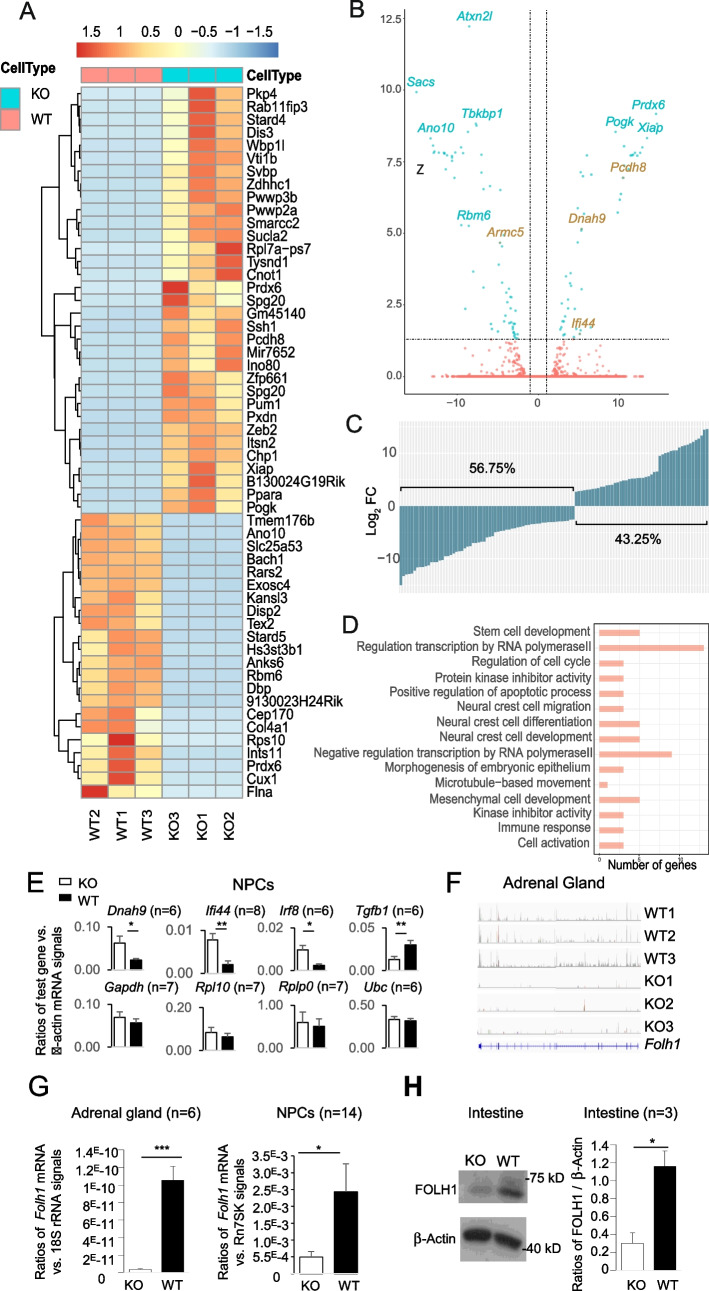
Transcriptome analysis of KO and WT NPC by RNA-seq. RNA-seq was conducted using three pairs of biological replicates of WT and KO NPCs. **A** Heatmap of 55 genes with the lowest FDRs among 106 genes with FDR < 0.05 in NPC RNA-seq. Results of 3 biological replicate pairs (WT and KO) of NPCs are presented. Color represents the standard deviation beyond the normalized mean of each gene in all the samples tested (i.e., 3 WT and 3 KO NPC samples). **B** A volcano plot depicting fold changes and FDRs of mRNA levels of expressed genes in KO versus WT NPCs according to RNA-seq. The dashed vertical lines indicate log_2_ fold changes (twofold increase or decrease), and the dashed horizontal line marks FDR = 0.05. The names of some prominently changed genes with the lowest FDR or biggest fold changes are indicated. **C** Log_10_ fold changes of all the 111 transcripts from 106 genes with FDR < 0.05. The percentages of transcripts with upregulation and downregulation are shown. **D** GO analysis in terms of biological processes for the genes with FDR < 0.05. Fifteen terms with high relevance to NTD were selected out of 28 significant terms. The number of the FDR significant genes belonging to a particular term in the GO databank is indicated. More detailed information about this GO analysis, such as the *p*-values of the terms after *Bonferroni* correction and the names of genes associated with each term, are presented in SI-Table 2. **E** Nuclear run-on validation of four genes with significant increase and four genes without change in the RNA-seq of KO NPCs. The number of repetitions is indicated. **F** Reduced *Folh1* RNA-seq read density in KO adrenal glands. *Folh1* RNA-seq read density tracks of three pairs of WT and KO adrenal glands are illustrated. **G** Reduced *Folh1* mRNA levels (mean ± SD) in the KO adrenal glands (left panel) and NPCs (right panel) according to RT-qPCR. The number of repetitions is shown. **H** Decreased FOLH1 protein levels in the intestine of KO mice according to immunoblotting. A representative blot is shown. The relative ratios (mean ± SD) of FOLH1 versus β-actin signals of KO and WT intestines based on three independent experiments are shown in the bar graph. *p < 0.05; **p < 0.01; ***p < 0.001 (paired two-way Student’s *t* tests)

Due to concerns that the abnormal Pol II pool size might systematically skew all the transcribed genes, we employed *Rn7sk* RNA as an internal control to normalize each sample’s reads. *Rn7sk* is transcribed by Pol III and is not subjected to the possible influence of Pol II [67, 68]. Indeed, *Rn7sk* expression in both KO and WT NPCs was similar (Additional file 1; Fig. S1). A threshold for transcript-level significance of FDR < 0.05 was applied to the paired comparison of RNA-seq results from 3 KO and 3 WT NPC biological replicates. After filtering out transcripts that were not true positives (true positives were defined as having a complete exact match of intron chains with a GffCompare class code of “ = ” [69]), we obtained 111 transcripts from 106 unique genes that showed significantly different expressions between KO and WT NPCs. These transcripts and genes are listed in Additional file 1, Table S1, along with their FDRs, fold changes, and read numbers. It is to be noted that three genes (i.e., *Fam172a*,* Slx1b*, and *Slc25a53*) each had one upregulated transcript and one downregulated transcript (Additional file 1, Table S1). Two other genes (*Pogk* and *Spg20*) each had two transcripts, but both transcripts were upregulated. This resulted in 46 unique genes with upregulated transcripts and 63 unique genes with downregulated transcripts. Fifty-five transcripts in this list with the lowest FDRs are shown in a heatmap (Fig. 7A). In this heatmap, Cnot1 appeared twice: once as an increased transcript and once as a decreased one, probably reflecting up- and downregulation of different isoforms of this gene. A volcano plot illustrates the fold change and FDR of the significantly changed genes, with several prominently changed ones annotated (Fig. 7B). *Armc5* was among the downregulated ones, as expected.

One of the possible roles of POLR2A ubiquitination is to remove persistently stalled Pol II to allow transcription to resume in the case of DNA damage or cellular stress. Failure to remove the stalled Pol II is believed to cause a general decrease in transcription. However, to our surprise, there was no generalized depression of transcription in the KO NPCs according to RNA-seq. As mentioned above, only 111 transcripts from 106 unique genes were significantly dysregulated, 48 (43.2%) being upregulated and 63 (56.8%) downregulated (Fig. 7C). For the vast majority of the genes (16,475–106 = 16,369 genes) that had detectable expression in NPCs, their expression was not influenced by the failed degradation of Pol II.

To understand the roles of those dysregulated genes in NTD pathogenesis, we performed a gene ontology (GO) analysis of the significantly changed genes for their relationship to biological processes. Twenty-eight significant terms were identified. In addition, eight terms with high relevance to neural tube development were also chosen, even though they were not statistically significant. The GO terms, term *p*-values corrected with Bonferroni step down, the number of genes associated with the terms, and the name of the associated genes are presented in Additional file 1, Table S2. Fifteen terms with known relevance to NTD were selected, and the number of the significant genes related to a particular term is depicted in a bar graph (Fig. 7D). This GO analysis will facilitate our future investigation regarding which and how the dysregulated genes cause NTD.

The steady-state mRNA levels determined by RNA-seq reflect a combined outcome of mRNA transcription and degradation. To ascertain that the mRNA upregulation according to RNA-seq of KO NPCs was genuine but not due to decreased mRNA degradation, we conducted nuclear run-on assays on several genes (i.e., *Dnah9*, *Ifi44*, *Irf8e7*, and *Tgfb1*) that were upregulated according to RNA-seq at the transcript level (*Dnah9* and *Ifi44*) or at the gene level (*Irf8* and *Tgfb1*). We confirmed that their de novo transcription was indeed upregulated, consistent with their steady-state mRNA levels according to RNA-seq (Fig. 7E). The nuclear run-on assay was also used to assess another group of four genes (*Gapdh, Rpl10, Rplp0*, and *Ubc*) that were not modulated in the KO NPCs according to RNA-seq. As expected, their de novo transcription was similar to their WT counterparts. These results corroborate that of RNA-seq and indicate that the RNA-seq results largely reflect the transcription rates in our experiments.

NTD has multifactorial pathogenic mechanisms, and dysfunctional NPCs probably only contribute to some extent to the pathogenic process. Other critical contributing factors include folate intake and metabolism [8]. Genes involved in folate metabolism have their prominent expression in tissues other than NPCs or neural tubes. In a separate project where we conducted an RNA-seq of the adrenal glands, we noticed that *Folh1* expression in the KO tissue was significantly reduced (Fig. 7F). This finding was confirmed by RT-qPCR (Fig. 7G, left panel). In NPCs, the *Folh1* mRNA level was too low to be detected by RNA-seq, but RT-qPCR showed significantly lower *Folh1* expression in the KO NPCs (Fig. 7G, right panel). More importantly, the FOLH1 protein level in the KO intestine was significantly reduced, the intestine being the site where FOLH1 exerts its function in folate absorption (Fig. 7H).

This transcriptome study indicates that in the KO NPCs, there is no generalized transcription suppression or upregulation. A subgroup of genes in NPCs and the intestine from KO mice are dysregulated. Some of these dysregulated genes have functions relevant to neural tube development and might contribute to NTD pathogenesis.

### The effect of compromised POLR2A degradation on gene-associated Pol II peak density

**Fig. 8:**
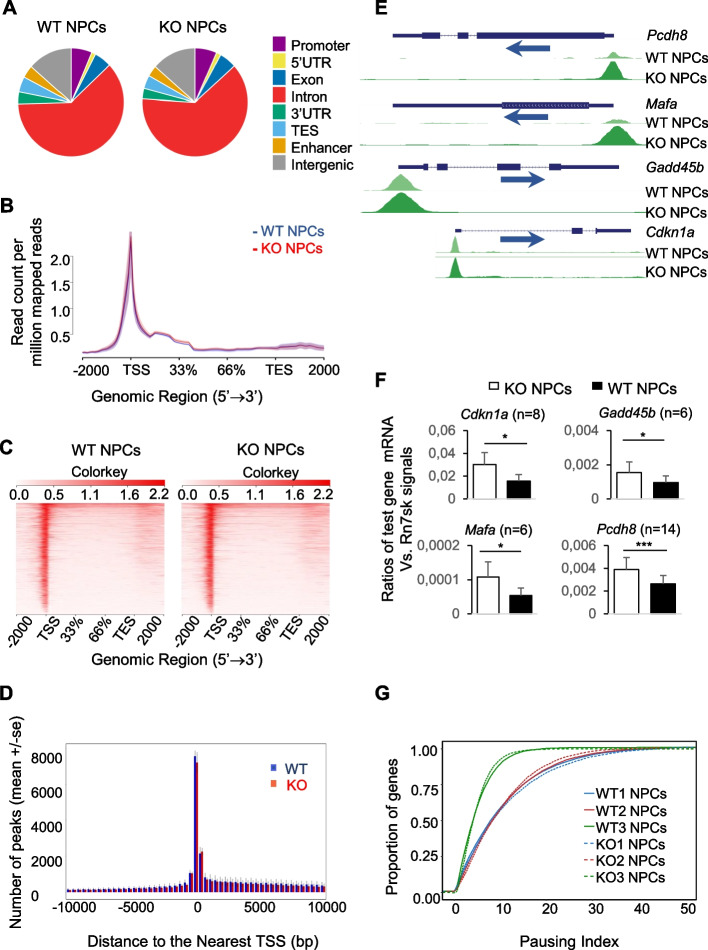
POLR2A ChIP-seq analysis of KO and WT NPCs. ChIP-seq was conducted using three pairs of biological replicates of WT and KO NPCs. **A** Pol II peak distribution in different gene regions of a representative pair of KO and WT NPC samples. **B** Means (solid lines) ± SE (shadows) of normalized read counts (read counts per million mapped reads) in a metagene analysis for a genomic region from − 2 kb of TSS to + 2 kb of TES. **C** Heatmaps of counts per million reads based on data from a representative pair of KO and WT NPC samples. **D** Pol II peak distribution (means ± SE) in a fixed region from − 10 kb upstream to + 10 kb downstream of TSS. **E** POLR2A read count tracks in the genes of *Cdkn1a*,* Gadd45b*,* Mafa*, and *Pcdh8*. The tracks were normalized so that each value was proportional to the read count per base pair per 10 million reads. **F** Upregulated mRNA levels of the four genes with elevated Pol II peak density in their genes according to RT-qPCR. The number of repetitions is indicated. *p < 0.05; ***p < 0.001 (paired two-way Student’s *t* tests). **G** Pausing indices (PI) of genes with detectable signaling in ChIP-seq*.* The proportions of genes with different PI are plotted. Solid lines: WT NPC samples; dashed lines: KO NPC samples. No statistically significant difference in PIs between the KO versus WT NPC samples for all the genes with ChIP-seq signals was found (paired two-way Student’s *t* tests followed by multiple-testing correction)

The accumulation of Pol IIs in the KO cells raised the question of whether they were part of the stalled Pol IIs due to failed degradation. To answer this question, we conducted POLR2A ChIP-seq in NPCs, and the results were analyzed along with RNA-seq data. A total of 12,107 genes had discernable ChIP-seq signals. The ChIP-seq dataset is available in GEO (accession number: GSE169582) [70]. The distribution of Pol II peaks in different regions of the genome is illustrated in Fig. 8A. In both KO and WT NPCs, the introns had the highest peak number, followed by intergenic regions and promoter regions. Within the genes, the highest normalized *Polr2a* read counts (read count per million (CPM) of mapped reads) were accumulated near TSS (Fig. 8B). Representative counts per million heatmaps for the region from − 2000 bp upstream of TSS to + 2000 bp downstream of TES of all genes in one pair of WT and KO samples are illustrated in Fig. 8C. The Pol II peak density of all the genes for a fixed region spanning from − 10 kb to + 10 kb surrounding the TSS is shown in Fig. 8d. These metagene analyses (Fig. 8B–D) show no visible Pol II peak density differences between KO and WT NPCs. Such metagene analyses are for visual appreciation but are not suitable for statistical analysis.

However, statistical analysis revealed 59 individual genes with highly differential Pol II peak density (FDR < 0.1) in KO versus WT NSCs (23 genes in the TSS region (from TSS − 400 bp to TSS + 100 bp); 33 genes in the gene body region (from TSS + 100 bp to TES − 100 bp); and three genes in the TES region (from TES − 100 bp to TES + 2000 bp) (Additional file 1: Table S3). Interestingly, except for three genes (i.e., *Tex14, Ttyh1*, and *Adcyap1r1*), most of these genes (56 out of 59 genes) presented significantly increased peak density. The Pol II peak density tracks of 4 genes (i.e.,* Cdkn1a*,* Gadd45b*,* Mafa*, and *Pcdh8*) that had the highest increase of Pol II peak density in the KO NPCs or had known relevance to NTD were illustrated (Fig. 8E). The higher Pol II peak density in these genes was accompanied by increased mRNA levels, as confirmed by RT-qPCR (Fig. 8F). This finding has raised an interesting possibility that for a subset of genes, a larger Pol II pool might promote their transcription. We determined the pausing indices (PI) of KO and WT ChIP-seq results. PI is defined as the ratio of *Polr2a* CPM in the TSS region versus that in the gene body region of all the genes with ChIP-seq signals and is an indicator of the transcription activity. Genes in the KO and WT NPCs had no significant differences in PI (Fig. 8G), suggesting that there is no genome-wide Pol II pausing, and this is consistent with the RNA-seq results.

### ARMC5 mutation was a risk factor for human NTD

Having demonstrated the involvement of ARMC5 in mouse NTD, the logical next step was to study whether *ARMC5* mutation was relevant to human NTD. We recruited a cohort of 511 MM patients, MM being a severe form of NTD. Single-nucleotide variants (SNVs) in *ARMC5* transcripts of these MM subjects were assessed by whole-exome sequencing. Among the 511 MM subjects, 257 were Americans of European descent, and 254 were Mexican-Americans. The control populations of Non-Finnish Europeans and Ad Mixed Americans in the genome aggregation database (gnomAD) were used as reference controls. These controls were not selected for or against MM.

The MM subjects’ age, alternate SNV position and protein mutation, alternate allele counts, allele number, and allele frequency are shown in Table 1. The allele numbers varied for different SNVs in both MM subjects and controls due to differences in exon-capturing techniques and batch effect. Larger allele number variation occurred in gnomAD controls because the data were compiled from multiple projects.

**Table 1:** ARMC5 SNVs in myelomeningocele patients

Subject code	Gender	Pre-FA fortification	Mutation ID	Protein mutation	rs ID (dbSNP151)	Adjusted AC (EUR)	Adjusted AN (EUR)	AF (EUR)	Control AC (NFE)	Control AN (NFE)	Control AF (NFE)	OR	*p*-value	CADD phred
B39-407	M	1998	16:31,469,751:A:G	p.Thr12Ala	rs979451735	1	510	0.20%	0	18,978	0.00%	¥	0.026	13.12
C42-1019	F	1960	16:31,470,942:C:T	p.Pro128Ser	rs200309618	1	450	0.22%	32	41,112	0.08%	2.86	0.302	19.13
B34-267	M	1993	16:31,473,868:C:T	p.Arg429Cys	rs539440145	1	494	0.20%	0	42,506	0.00%	¥	0.011	24.1
E75-466	F	1985	16:31,477,780:G:A	p.Arg888Gln	rs199498431	1	470	0.21%	19	42,368	0.04%	4.75	0.198	22.8
**Subject code**	**Gender**	**Pre-FA fortification**	**Mutation ID**	**Protein mutation**	**rs_dbSNP151**	**Adjusted AC (MEX)**	**Adjusted AN (MEX)**	**AF (MEX)**	**Control AC (AMR)**	**Control AN (AMR)**	**Control AF (AMR)**	**OR**	***p*-value**	**CADD phred**
A23-456	F	1991	16:31,470,793:G:A	p.Arg78His	rs920446902	1	500	0.20%	2	13,094	0.02%	13.12	0.106	27.5
D68-941	M	2004	16:31,470,811:C:T	p.Ala84Val	rs202112554	1	500	0.20%	13	14,204	0.09%	2.19	0.384	13.5
D46-697	M	1989	16:31,474,085:G:A	p.Arg501Gln	rs749775865	1	500	0.20%	3	17,048	0.02%	11.39	0.109	21.2
C84-353	F	1998	16:31,474,132:G:A	p.Gly517Ser	rs372472557	1	498	0.20%	2	17,048	0.01%	17.15	0.083	23
F78-368	M	1986	16:31,476,020:C:T	p.Pro654Leu	rs200115942	1	488	0.20%	7	16,780	0.04%	4.92	0.205	20.6

A total of 511 subjects from North America were selected for WES. Rare ARMC5 variants with C-scores > 13 are presented. Protein mutation is presented with amino acid position numbered according to the longest 1030-aa ARMC5 isoform c (ENSP00000386125 or NP_001275696). Mutations at positions 12, 78, and 84 are not present in the most abundant 935-aa isoform (ENST00000268314.4 or NP_001098717). *EUR* European descent, *Mex* Mexican–American, *NFE* non-Finnish European, *AMR* Ad Mixed American, *AC* allele count, *AN* allele number, *AF* allele frequency, *OR* odds ratio, *CADD Phred* Combined Annotation Dependent Depletion Phred score (C-score)

**Fig. 9:**
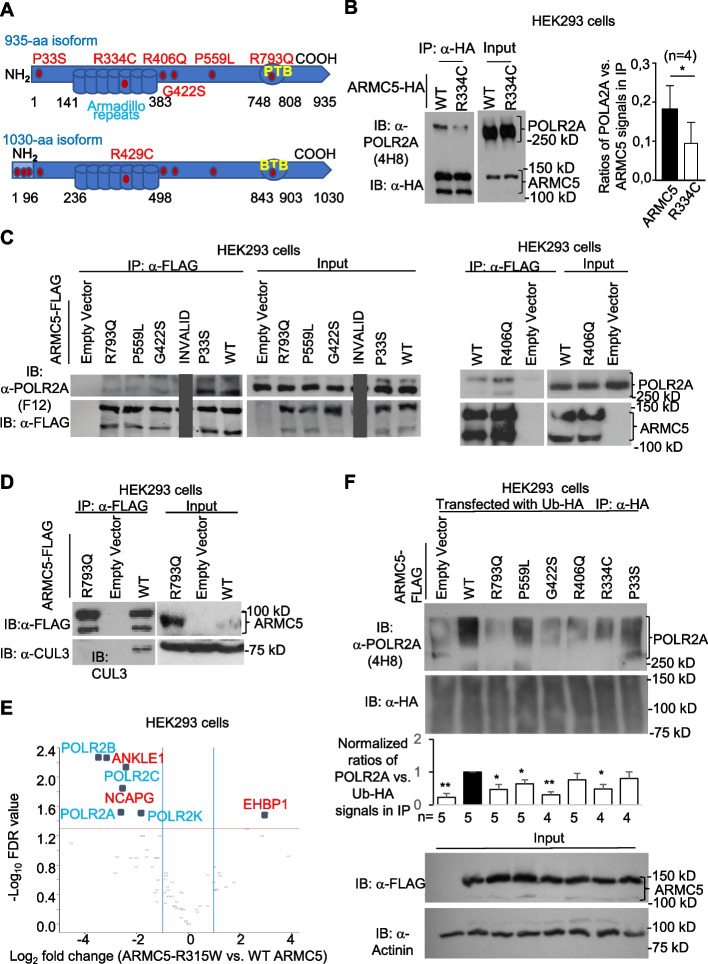
ARMC5 with mutations found in the MM and PBMAH patients had compromised affinity and function. **A** A schematic illustrating the ARMC5 protein structure with mutations (red dots) found in MM patients. The upper schematic shows the most abundant 935-aa ARMC5 isoform, and the mutation positions are numbered according to this isoform. The lower schematic shows the longest 1030-aa isoform, with 95 extra aa in the N-terminus. The R429C mutation in this long isoform is the same as the R334C mutation in the 935-aa isoform. **B** Reduced binding between ARMC5 R334C and POLR2A in HEK293 cells. HEK293 cells were transfected with plasmids expressing WT ARMC5-HA or ARMC5 R334C mutant-HA. The association of WT and mutant ARMC5 with endogenous POLR2A was detected according to anti-HA Ab immunoprecipitation followed by anti-POLR2A Ab (4H8) immunoblotting. **C** Reduced binding of ARMC5 R793Q, P559L, and G422S with POLR2A in HEK293 cells. HEK293 cells were transfected with constructs expressing FLAG-tagged WT or mutant ARMC5s. An empty vector was used as an additional control. The association of WT and mutant ARMC5 with endogenous POLR2A was detected according to anti-FLAG Ab immunoprecipitation followed by anti-POLR2A Ab (F12) immunoblotting. **D** Reduced binding between ARMC5 R793Q mutant and endogenous CUL3. HEK293 cells were transfected with constructs expressing FLAG-tagged WT ARMC5 or ARMC5 R793Q mutant. The association of WT or mutant ARMC5 with the endogenous CUL3 was detected according to anti-FLAG Ab immunoprecipitation followed by anti-CUL3 Ab immunoblotting. **E** A volcano plot showing reduced binding between the FLAG-ARMC5 R315W mutant (a mutation found in PBMAH patients) expressed in HEK293 cells and POLR2A according to anti-FLAG Ab immunoprecipitation followed by LC-MS/MS. The horizontal line: FRD = 0.05, based on three biological replicates. The vertical lines: + 2- and − twofold changes. **F** Reduced POLR2A ubiquitination in HEK293 cells expressing 4 mutant ARMC5s. HEK293 cells were transfected with constructs expressing FLAG-tagged WT or mutant ARMC5s along with plasmid expressing ubiquitin-HA. An empty vector was used as an additional control. Ubiquitinated POLR2A was detected by anti-HA precipitation followed by anti-POLR2A Ab (4H8) blotting. Signals of all ubiquitinated proteins in the precipitates (second row) were used to monitor the immunoprecipitation efficiency. The signals of ARMC5 (detected by anti-FLAG Ab) and α-actinin in the lysates were used to confirm similar ARMC5-FLAG expression and input protein quantity, respectively, in each sample. Bar graphs in **B** and **F** summarize the results from more than 3 repetitions, using the ratios of densitometry signals of POLR2A (**B**) or ubiquitinated POLR2A (**F**) versus their respective controls (ARMC5 for **B** and Ub-HA for **F**). *p < 0.05; **p < 0.01 (paired two-way Student’s *t* tests)

The deleteriousness of the SNVs in *ARMC5* of the MM subjects was calculated according to the Combined Annotation Dependent Depletion Phred score (C-score) [71]. Nine SNVs representing the top 5% of most deleterious ones (i.e., with C-score > 13.01) found in *ARMC5* transcripts of MM subjects are listed in Table 1. These SNVs were all missense variants, and their positions are illustrated in Fig. 8a. ARMC5 has several isoforms, all coded by the same *ARMC5* gene. The 935-aa isoform (NP_001098717.1) (upper panel, Fig. 9A) is the most abundant one presented in almost all the tissues [18]. The 1030-aa isoform (NP_001275696.1) (lower panel, Fig. 9A) is the longest and has a 95-aa N-terminal region coded by two extra exons, but this isoform is only present in a few tissues at a relatively low level [18]. The longest ARMC5 isoform was used to number the SNV positions in Table 1 so that all the mutations in any isoform can be presented in the table. Using the longest isoform for the position numbering purpose does not mean that these SNVs only exist in this longest isoform. In all likelihood, most of these SNVs, except for the three in the first 95-aa region in the N-terminus, are in the most abundant 935-aa isoform. The positions of these mutants numbered according to the longest 1030-aa isoform are also translated to positions according to the 935-aa isoform in Fig. 9A. Later, our functional validation of these mutants would use the 935-aa isoform-based numbering.

These nine SNVs were rare ones (defined as having allele frequency < 0.01), four and five being in European-American and Mexican–American MM subjects, respectively. Two of the four variants in European-American MM subjects and four of the five variants in Mexican–American MM subjects were assigned as the top 1% deleterious variants (C-scores > 20). Two variants (p.T12A rs979451735 and p.R429C rs539440145) in European-American MM subjects were not present in gnomAD non-Finnish European controls, and they were considered novel SNVs. Their alternate allele counts were significantly higher than those of the Non-Finnish European controls (*p* < 0.05). The *p*-value of the alternate allele count of one rare SNV found in Mexican–American MM subjects was approaching significant (*p* = 0.083). Since all variants identified in the approximately 9-kb *ARMC5* loci were in linkage disequilibrium, these *p*-values were not subjected to a multiple-testing penalty.

The results from this genetic study confirm that, indeed, ARMC5 mutation is significantly associated with NTD risks in humans. Our current genetic materials do not allow us to determine whether these SNVs in ARMC5 are de novo or inherited ones.

### Human ARMC5 mutations compromise the activity of the ARMC5-containing E3

Having found nine deleterious ARMC5 SNVs in MM patients, we needed to ask whether these SNVs affected the E3 activity. Three SNVs were located only at the N-terminus of the longest isoform. The most significant R334C mutation (position based on the 935-aa isoform, or position R429C based on the 1030-aa isoform) was in the 5th repeat in the ARM domain. One SNV was at the N-terminus before the ARM domain in the 935-aa isoform. Three SNVs (i.e., R406Q, G422S, and P559L) were scattered along the region between the ARM domain and the BTB domain. One SNV(R793Q) was found in the BTB domain (upper panel, Fig. 9A). According to our previous deletion mutation studies [45], the ARMC domain is critical for POLR2A binding. The region before the ARM domain (aa 1–141 based on the 935-aa isoform), the region between the ARM domain, and the BTB domain also contribute to POLR2A binding but to a lesser extent [45]. We thus investigated how the mutations in these domains and regions affected the ARMC5 binding to POLR2A and CUL3. Currently, we have no knowledge about the function or the possible binding partners of aa 1–96 in the N-terminal region (positions according to the 1030-aa isoform) in the 1030-aa ARMC5 isoform (lower panel, Fig. 9A). It is not feasible to study the binding of the three SNVs with their unknown partners.

When transfected into HEK293 cells, the ARMC5 R334C mutant co-precipitated significantly less POLR2A compared to WT ARMC5 (Fig. 9B), indicating that this mutation hampered the interaction between ARMC5 and POLR2A. Similarly, ARMC5 with mutations at G422S, P559L, and R793Q showed reduced binding with POLR2A (Fig. 9C). However, ARMC5 mutations P33S and R406Q minimally affected POLR2A binding, suggesting that they are not essential for substrate recognition.

CUL3 has a BTB-interacting domain, and it recruits a BTB domain-containing protein as its substrate recognition unit to form an active E3 [60]. The ARMC5 BTB domain is critical for the association with CUL3, according to our previous study [45]. ARMC5 R793Q mutation in the BTB domain resulted in a decreased association with CUL3 (Fig. 9D).

ARMC5 R315W mutation (position based on the 935-aa isoform) is significantly associated with the PBMAH [45]. This mutation is also located in the 5th repeat in the ARM domain. We tested ARMC5 R315W for its binding with POLR2A using a different approach, i.e., immunoprecipitation followed by LC–MS/MS. The proteomics dataset is available in proteomeXchange (accession number PXD047572) [72]. This mutant ARMC5 had about a fourfold lower binding capacity to POLR2A than that of WT ARMC5 (FDR < 0.05) (Fig. 9E). It is interesting to note that in addition to POLR2A, the binding of the ARMC5 R315W mutant to other components of Pol II, such as POLR2B, POLR2C, and POLR2K was also reduced (Fig. 9E). This suggests the association of WT ARMC5 with these Pol II subunits (whether directly or via POLR2A is yet to be determined) and a reduced association due to the mutation.

We asked whether these ARMC5 mutations compromised the E3 function. HEK293 cells were transfected with WT or mutant ARMC5, along with HA-tagged ubiquitin. The ubiquitination of the endogenous POLR2A was determined by anti-HA immunoprecipitation of de novo ubiquitinated proteins, followed by anti-POLR2A immunoblotting. As shown in Fig. 8f, the mutations in ARMC5 at R334C, G422S, P559L, and R793Q all caused a significant reduction of POLR2A ubiquitination compared to that of WT ARMC5. Consistent with the result of the binding study, in which the ARMC5 P33S and R406Q mutations had no impact on POLR2A binding, these mutations did not alter POLR2A ubiquitination (Fig. 9F).

These binding and ubiquitination studies demonstrate that four of the six variants tested are functional ones affecting the POLR2A-specific E3 activity, corroborating the implication of POLR2A in MM pathogenesis. The remaining two variants are likely false positives in our human MM genetic studies.

## Discussion

We reported here that *Armc5* deletion in mice significantly augmented NTD risks. We discovered that ARMC5, being a substrate recognition subunit of a novel POLR2A-specific E3, was responsible for the degradation of almost all the 12 Pol II subunits, and its deletion caused a bona fide enlarged Pol II pool. ARMC5 KO only selectively influenced the transcription of 106 genes in NPCs, some of which, such as *Folh1*, are known to be involved in processes critical for neural tube development. Our human genetic study confirmed that ARMC5 mutations are modifiers of human NTD risks, and four variants were validated as functional ones.

### ARMC5-CUL3-RBX1 is a novel POLR2A-specific E3 and is responsible for the degradation of most Pol II subunits

Several POLR2A-specific E3 in mammalian cells have been reported, such as Nedd4 [67], Wwp2 [38], VHL/ElonginBC/Cul3/RBX1 [41, 68], and ElonginA/ElonginBC/Cul5/RBX2 [69]. These POLR2A-specific E3s have demonstrable activities after massive DNA damage and cellular stress caused by irradiation or chemicals when there is an excessive need to remove stalled Pol II. The activity of pVHL-EloB/EloC-CUL2-RBX1 and WWP2 can be detected in the absence of exogenous DNA damage in cell lines [38, 41] but has not been extended to tissues or organs. None of the previously identified POLR2A-specific E3s are involved in the degradation of the other Pol II subunits, with the exception of pVHL-EloB/EloC-CUL2-RBX1, which acts on POLR2G [44]. Theoretically, there must be an E3 (or E3s) in tissues and organs under physiological conditions, i.e., without artificially induced DNA damage or cellular stress, to degrade all the Pol II subunits. Such E3s are needed to remove misassembled Pol II, misfolded Pol II subunits, permanently paused Pol II (to resume transcription), or DNA-bound Pol II during DNA replication (to resolve collision between the transcription and DNA replication machinery during the S phase). Our study has discovered an ARMC5-based novel E3 that is responsible for the degradation of almost all the 12 subunits of Pol II under a physiological condition.

It is to be noted that after ARMC5 deletion, the protein accumulation of one of the Pol II subunits, POLR2C, had not reached significance, and one subunit, *Polr2h*, presented a moderate but significant increase (about twofold) in its mRNA levels in MEFs. However, the tendency of POLR2C protein increase is consistent, and a larger sample size will probably make the increase significant. On the other hand, the twofold mRNA increase seen in *Polr2h* is at best of marginal interest when we analyze RNA-seq or RT-qPCR data. Therefore, it is almost certain that this E3 is the major ubiquitin ligase that controls the degradation of all 12 Pol II subunits. Other POLR2A-specific E3s may be needed only when there are excessive demands, such as significant DNA damage or severe cellular stress.

The effect of ARMC5 on the degradation of all 12 Pol II subunits can be achieved in two possible ways. First, these subunits might directly associate with ARMC5 and are additional substrates of this novel E3. While we cannot totally rule out this scenario, a more likely possibility is that the other 11 Pol II subunits are brought to the vicinity of this E3 via the interaction of ARMC5 and POLR2A. In supporting this latter hypothesis, we previously demonstrated the direct interaction between ARMC5 and POLR2A by yeast 2-hybrid assay [19], while none of the other Pol II subunits were identified in that assay. The result of both possibilities is the same: in the absence of ARMC5, this E3 is not functional, leading to compromised degradation of all Pol II subunits and, hence, a bona fide enlargement of the Pol II pool under physiological conditions. The effect of the E3 on all the Pol II subunits, particularly POLR2D and PLOR2G, which are quite distant from POLR2A, raises interesting possibilities that this E3 might not only act on Pol II but other nearby components of the transcription machinery, such as the preinitiation complex (PIC) or the Mediator complex. Studies in these aspects are ongoing.

According to ChIP-seq, the majority of genes with differential peak density (56 out of 59) had increased gene-binding POLR2A in KO NPCs. Although POLR2A was used as a surrogate marker of Pol II in the assay, the accumulation of all the Pol II subunits in the KO cells suggests that increased POLR2A peak density represents an enlarged functional gene-associating Pol II pool. The accumulated Pol IIs were prominently located in the TSS region, raising an intriguing possibility that under physiological conditions, one arm of the dimeric E3 binds one Pol II complex via POLR2A while the other empty arm of this dimeric E3 wraps around the Pol II complex, using its catalytic RBX1 core to ubiquitinate other Pol II subunits, PIC components, or Mediator subunits.

The augmented Pol II peak density in some genes in the KO cells raises other interesting questions: does this E3 play a role in resolving the replication-transcription conflict under a physiological condition, and does its absence hamper the conflict resolution, leading to slower proliferation? Indeed, we observed that different types of KO cells we tested so far, such as NPCs in this study, T cells in our previous study [19], and MEFs (unpublished data), all have reduced proliferation. This possibility is under active investigation.

It will be prudent to state that in addition to Pol II, this ARMC5-CUL3-RBX1 E3 might have other substrates in addition to those in the transcription machinery and even in the cytosol, and the dysfunction such substrates after *Armc5* KO or mutation contributes to some of the observed phenotypes in the KO mice and patients with ARMC5 mutations. The other ARMC5-binding partners found by yeast 2-hybrid assay [19] and ARMC5 immunoprecipitation (Figs. 3A and 9E) are good candidates for future validation studies.

### The effect of compromised Pol II degradation on the transcriptome

During mRNA transcription, if the transcription machinery encounters template DNA damage or cellular stress, Pol II will stall until the damage is repaired or the stress relieved [45]. It is believed that persistent Pol II stalling prevents transcription from resuming unless the stalled Pol II is degraded by proteasomes [28, 29, 73]. It follows that if POLR2A ubiquitination is compromised, there will be a general decrease in mRNA transcription. We assessed the transcriptome of KO NPCs by RNA-seq, but to our surprise, only 63 genes out of the 16,475 expressed genes in the KO NPCs showed reduced mRNA levels. We validated RNA-seq results in selected genes by the nuclear run-on assay, which measures the transcription rate. The results were compatible with their steady-state mRNA levels, suggesting that the steady-state mRNA levels determined by RNA-seq largely reflected the transcription rates. The pausing index, which is the ratio of Pol II density in the promoter region versus that in the gene body, is often used to gauge the transcription activity of a gene [74, 75]. However, the pausing indices between the KO and WT NPCs had no significant difference (Fig. 7G). These results collectively indicate no general decrease in the transcription rate in the KO cells despite the failed POLR2A degradation.

These findings suggest two non-competing possibilities. It is possible that Pol II stalling is an insignificant event under a physiological condition. Even without this dominant E3, some other Pol II-specific E3s are sufficient to remove the small amount of stalled Pol II. Consequently, transcription is not systemically compromised. Equally possible is that our current knowledge about removing stalled Pol II by proteasomes is based on experiments using cells with massive DNA damage [76] or based on in vitro experiments [77, 78]. Maybe the ubiquitination and proteasome-mediated Pol II degradation are not needed to remove the stalled Pol II at all, and there are other mechanisms to recycle stalled Pol II. Indeed, this is the case in yeasts [79, 80]. A recent computer modeling study shows that Pol II can come off the damaged DNA template and recycle instead of being degraded [30]. The end result of both scenarios is the same: the loss of the major Pol II-specific E3 does not cause generalized Pol II stalling under a physiological condition.

One of the functions of this E3 is likely to control the Pol II pool size. How the Pol II pool size affects transcription and cellular function is a question infrequently visited. Intuitively, we would believe that since the same Pol II works for all the genes, its pool size shall universally affect all of them and probably increase their transcription. However, this is not the case. The accumulation of POLR2A due to *Armc5* KO in NPCs only selectively influenced a limited number of genes (46 upregulated and 63 downregulated).

How does Pol II pool size affect the expression of a subset of genes? First, it does not work alone but needs additional tissue-specific transcription factors to modulate the transcription rate jointly. Recently, Vidakovic reported that an enlarged Pol II pool due to POLR2A K1268R mutation results in the upregulation of more than 1600 genes in HEK293 cells but the downregulation of only a few hundred genes [30]. We similarly observed predominantly upregulated genes in the KO adrenal glands [45]. The abnormally expressed genes in these different cell types (NPCs (our current RNA-seq data), HEK293 cells [30], and adrenal glands [45]) showed vast differences. Likely, the enlarged Pol II pool and tissue-specific transcription factors jointly influence gene expression. The exact mechanism is unknown, but we can offer some speculations.

For the upregulated genes, their promoters might contain more Pol II docking motifs, favoring more active transcription if the Pol II pool is enlarged. In supporting this hypothesis, most ChIP-seq significant genes had upregulated Pol II peak densities (56 out of 59 genes; SI-Table 3). Higher Pol II peak density was found in the promoter region in many upregulated genes (Fig. 8E). It is to be noted that among the 56 genes with significantly increased POLR2A peak density, only five had increased mRNA levels according to RNA-seq in the KO NPCs. How do we explain the lack of significant overlap of genes with both upregulated POLR2A peak density and upregulated mRNA? We conducted RT-qPCR of a selected group of genes with upregulated POLR2A peak density but without mRNA increase according to RNA-seq. As shown in Fig. 7F, with a larger number of biological replications (*n* = 6 to 14), mRNA upregulation became significant in the KO NPCs, indicating that they were false negatives in RNA-seq. The inadequate statistical power due to a small number of biological replications (*n* = 3) used in RNA-seq combined with the low difference between the KO and WT cells might be the reasons for false negatives. Conceivably, RT-qPCR validation with a larger number of biological replications might find more overlap between the genes with higher Pol II peak density and genes with upregulated mRNA.

It is more difficult to understand why a larger Pol II pool causes the downregulation of some genes. It is possible that some of these downregulated genes are indirectly controlled by the larger Pol II pool via some upregulated ones or are regulated by other substrates of this E3.

### ARMC5 mutations as an NTD risk modifier

In *Armc5* KO mice, 26% of KO fetuses/mice suffered from defective neural tube development. Since NTD penetrance in the KO mice was not 100%, it means that the *Armc5* mutation alone is not sufficient to cause NTD. Our mice were in a C57BL/6 × CD1 background. We previously described the phenotype of the *Armc5* KO mice in the C57BL/6 background, in which NTD was not obvious [19]. Nakazawa et al. generated mice in the C57BL/6 background with POLR2A K1268R knock-in [31], which caused defective POLR2A degradation and hence POLR2A accumulation. NTD was not reported in those knock-in mice either. We noticed that WT mice with a 50% CD1 genetic background were more prone to develop NTD because 3.7% of the WT mice in the CD1 x C57BL/6 background manifested kinky tails, while 0% of the mice in the pure C57BL/6 background did so. This indicates that *Armc5* is an NTD risk modifier, and its mutations need to interact with other genetic factors, such as those in the CD1 genetic background, to cause NTD.

The KO mice presented a small body size, which reflects abnormal bone development throughout the body, among other things. Is the kinky tail part of such abnormal bone development? Small body size frequently occurs in about 31% of the 1997 strains of viable knockout strains surveyed [81], but the vast majority of the 620 stains of small KO mice have no kinky tail phenotype. In the case of ARMC5 KO mice, when they were in the C57BL/b6 background, in spite of their small body size, they showed no kinky tails. This literature and observations from our laboratory indicate that kinky tails are not caused by abnormal bone development but rather a phenotype of NTD.

The reduced proliferation of KO cells likely contributed to NTD development in mice. The compromised KO NPC proliferation and cell cycle progression based on colorimetric assay and flow cytometry were evident. However, immunofluorescence Ki-67 staining of e9.5 neural plates with exencephaly failed to detect a significant difference in the KO and WT tissues. This is likely due to different assay sensitivities. Immunofluorescence assay (including Ki-67 staining) of tissue samples has much higher noise compared to colorimetric in vitro cell proliferation assays and flow cytometry. A lack of detection of reduced proliferation in KO tissue sections does not mean such reduction does not happen. In fact, the KO embryos, including their neural tubes, are much smaller than the WT counterparts. This is clear evidence that all the KO tissues have reduced proliferation in vivo.

Our human genetic study detected nine highly deleterious ARMC5 SNVs in MM patients. This finding confirms the relevance of our mouse data to human NTD. In humans, ARMC5 with four functionally validated SNVs caused the reduced binding to POLR2A and reduced ubiquitination of the latter, suggesting the role of POLR2A as an effector downstream of ARMC5 in causing MM, although further experiments are required to validate this supposition. The enlarged Pol II pool probably dysregulates actual culprit genes further downstream, such as *Cdkn1a*,* Gadd45b*,* Mafa*, and *Pcdh8*, critical for processes (e.g., proliferation and apoptosis) essential for proper neural tube development [82–86]. Our data clearly demonstrated that the ARMC5 was critical for Pol II homeostasis. However, the cause-and-effect relationship between an ARMC5 deletion/mutation-induced enlarged Pol II pool and NTD pathogenesis remains to be established, and further investigation of the putative culprit effector genes is required.

The NTD phenotypes of mice and humans were not identical. Several reasons can explain such differences. First, the null mutation occurred in mice in both alleles, and the KO mice had no functional ARMC5 protein. In contrast, in humans, the mutations were monoallelic, and some of them were point mutations. Therefore, some functional ARMC5 proteins were still present. Such quantitative differences may contribute to different NTD phenotypes in mice and humans. Secondly, 60% of KO embryos/fetuses died between e13.5 and 3 weeks of age. The survival ones were likely those with a milder phenotype. We have no information on how many premature deaths occurred in individuals with ARMC5 mutations (monoallelic or biallelic). Thirdly, humans and mice are two different species. Other genetic and environmental factors are needed to induce NTD, and these factors are quite different in mice and humans, causing different NTD phenotypes. As described in the result section, even KO mice in a different genetic background (i.e., C57BL/6) did not have an NTD phenotype.

*Armc5* KO or mutation can affect genes in other organs and tissues whose function might be indirectly needed for proper neural tube development. Reduced FOLH1 expression in the KO intestine is a case in point. *FOLH1* is a transmembrane protein and is a glutamate carboxypeptidase [87]. It is well established that sufficient folate is required for proper neural tube development [8]. Folate needs to be absorbed as an essential nutrient from food [8]. Dietary folate deficiency and dysfunction of folate absorption and metabolism increase NTD risks [9–11]. Dietary folate exists in a polyglutamate form and needs to be digested by *FOLH1* into monomers to be absorbed by the small intestine [88]. Homozygous *Folh1* KO in mice was embryonically lethal, indicating the vital function of FOLH1 in development [89, 90]. Several human studies showed that FOLH1 mutations are associated with low serum folate levels and increased NTD risks [87, 91, 92]. Thus, the reduced *Folh1* expression in the KO intestine, either due to failed Pol II degradation or the reduced function of mutant ARMC5 on other substrates, might contribute to the increased NTD risks.

Fetuses obtain foliate from their mothers via the placentas. Obviously, the dysregulated folate metabolism needs to occur in the mothers to cause NTD in the fetuses. So, the reduced expression of FOLH1 in the KO mice is not the direct cause of their own NTD. Rather, the ARMC5 haploinsufficiency in the mother of the KO mice or humans is likely an NTD risk modifier. Additional experiments are needed to prove this hypothesis and to fully establish the cause-and-effect relationship between the ARMC5 KO/mutation-induced low FOLH1 expression and NTD pathogenesis.

## Conclusions

Our findings revealed that *Armc5* KO or mutation was associated with NTD risks in mice and humans. ARMC5-CUL3-RBX1 was a novel dominant E3 controlling the degradation of all 12 Pol II subunits under physiological conditions. *Armc5* KO or mutation caused an enlarged Pol II pool. A subset of genes was dysregulated due to ARMC5 mutations, either as a consequence of the enlarged Pol II pool or due to failed degradation of other substrates of this E3. Among this subset of genes, some, such as FOLH1 expressed in the intestine, might be effector genes culpable for the pathogenesis of NTD. Further investigation is needed to determine the cause-and-effect relationship between an enlarged Pol II pool and NTD and to validate the potential role of downregulated FOLH1 expression in NTD pathogenesis.

## Methods

### In situ hybridization

To determine *Armc5* mRNA tissue-specific expression, we employed 1526-bp (starting from GATATC to the end) mouse *Armc5* cDNA (GenBank: BC032200, cDNA clone MGC: 36,606) in pSPORT1 as a template for sense and antisense riboprobe synthesis, using SP6 and T7 RNA polymerase for both ^35^S-UTP and ^35^S-CTP incorporation. Tissues from WT mice were frozen in − 35 °C isopentane and kept at − 80 °C until sectioned. X-ray autoradiography focused on 10-μm-thick cryostat-cut sections. Briefly, overnight hybridization at 55 °C was followed by extensive washing and digestion with RNase to eliminate non-specifically bound probes. Anatomical-level images of in situ hybridization were generated using X-Ray film autoradiography after 4-day exposure.

### RT-qPCR

Total RNA from cells or tissues was extracted with Rneasy kit (Qiagen) and reverse-transcribed with iScriptTM cDNA Synthesis Kit (Bio-Rad Laboratories). The primer sequences are listed in SI-Table 4. Rn7sk or β-actin was used as internal controls. The samples were first denatured at 95 °C for 2 min. They then underwent 40 cycles of amplification using the following cycling conditions: 95 °C for 15 s, 60 °C for 60 s, and finally, with a melting step from 72 to 95 °C for 5 s. qPCR signals between 22 and 30 cycles were analyzed. Samples were assayed in triplicate, and the data were expressed as signal ratios of target mRNA/internal control mRNA.

### ARMC5 KO mice

ARMC5 KO mice were described in our previous publication [19]. These mice were bred into the CD1 x C57BL/6 F1 background for this study.

### Micro-CT whole-body bone imaging

The mice were euthanized by CO_2._ The whole-body bone images were obtained by scanning the mice using Broker SkyScan1176 Micro-CT.

### Collection of mouse fetuses

Fetuses were harvested for neural tubes (e8.5, e9.5, and e10.5), for the assessment of exencephaly (e9.5 or e12.5), and for CNS tissues to generate NPCs (e13.5).

### Neural tube isolation

The neural tubes were isolated from e8.5 or e9.5 mouse embryos under a dissecting microscope and digested with pancreatin (6 mg/ml in PBS) for 6 min at room temperature. Sticky lateral tissues were teased away, and cleaned neural tubes were used in the experiments.

### Immunofluorescence

E9.5 embryos were fixed in PBS containing 4% paraformaldehyde at 4 °C overnight and then sequentially soaked in PBS containing 30% sucrose at 4 °C for 24 h, followed by a mixture of 30% sucrose (in PBS) and OCT at a 1:1 ratio at 4 °C for another 24 h. The samples were then embedded in OCT and stored at − 80 °C until use. Fetal WT and KO neural tubes at the level of hindbrains were cryosectioned (10–12 µm) transversely. The cryosections were first permeabilized with 0.3% Triton X-100 in PBS for 3 min and treated with blocking buffer (PBS containing 5% goat serum and 0.1% Tween 20) at room temperature for 1.5 h. To quantify apoptosis in e9.5 neural tubes, we assessed the cryosections by fluorescent TUNEL using an in situ Cell Death Detection Kit (Roche) according to the manufacturer’s instructions.

Fluorescent images were collected on an AxioPhot fluorescent microscope (Zeiss). The images were analyzed using the Cell Counter Plugin of the ImageJ software. TUNEL-positive cells among total cells, which were visualized by DAPI staining, in the neural folds and adjacent areas were registered.

For immunofluorescent staining of NPCs, the cells were cultured on poly-D-lysine and laminin pre-coated glass slips in the NeuroCult proliferation medium for 1 day. The cells were fixed with 4% paraformaldehyde in PBS and permeabilized with PBS containing 0.3% Triton X-100 for 3 min. The slips were then soaked in blocking buffer (PBS containing 5% goat serum and 0.1% Tween 20 at room temperature) for 1.5 h and reacted with different first Abs (mouse anti-Nestin mAb, 4 µg/ml, Abcam; rabbit anti-Sox2 Ab, 1 µg/ml, Abcam). The coverslips were then incubated with secondary Abs (AlexaFluor488-conjugated goat anti-mouse Ab; Invitrogen; rhodamine-conjugated goat anti-rabbit Ab; Jackson ImmunoResearch Laboratories) in the blocking buffer for 2 h at room temperature. The coverslips were washed three times with PBS and mounted in ProLong gold anti-fade containing DAPI (Invitrogen).

For immunofluorescent staining of cytosolic and nuclear ARMC5, SK-N-SH neuroblastoma cells were cultured on CELLstart substrate (Invitrogen)-pre-coated coverslips overnight and transiently transfected for 2 days with plasmids expressing human ARMC5-HA (Genecopoeia) using Lipofectamine 3000 transfection reagent (Invitrogen). The procedure of immunofluorescent staining was the same as that for NPC staining, except that rabbit anti-HA mAb (Cell signaling Technology) and rhodamine-conjugated goat anti-rabbit Ab (Jackson Laboratories) were used as the primary and secondary Abs, respectively.

### Generation of mouse NPCs

The brains from e13.5 mouse fetuses were separated at the cervical spinal cord level, and the ganglionic eminences were dissected and harvested. The harvested tissue pieces were collected in a complete neural stem cell medium (NeuroCult NSC Basal Medium and NeuroCult NSC Proliferation Supplements at a 9:1 ratio; Stemcell Technologies) and dissociated thoroughly but gently by pressing the pipette tip to the bottom of the tube and pipetting five times to obtain a single-cell suspension. The cells were plated at the density of 2 × 10^5^ cells/ml in a complete NSC medium supplemented with 20 ng/ml EGF; Stemcell Technologies). Five to 6 days later, the neurospheres were treated with Accutase (Stemcell Technologies) and cultured for additional 5–6 days. The neurospheres of the second passage were used for experiments.

### NPC proliferation assay

NPCs were cultured in 96-well plates in a complete NeuroCult proliferation medium for 1 day. CellTiter 96 AQueous One Solution (20 μl/well; Promega) was added to the wells. After an additional 2-h culture, the absorbance of the wells at 490 nm was registered with an ELISA reader.

### Flow cytometry

For cell cycle analysis, NPCs were blocked at the G1 phase by aphidicolin (Millipore Sigma;12 µM) for 8 h. The cells were released into the S phase by washing three times with NeuroCult basal medium and then incubated with a complete NeuroCult proliferation medium. NPCs were collected at 0, 2, 4, 6, 8, 10, and 24 h later, fixed with 70% ethanol, and stained with propidium iodide for cell cycle analysis by flow cytometry.

For apoptosis analysis, NPCs were cultured without EGF or with different concentrations of EGF for 20 h. A single-cell suspension was obtained by treating the cells with ACCUTASE. The cells were stained with Annexin V (1:50 dilution; BD Bioscience) and analyzed by flow cytometry.

### LC–MS/MS

HEK293 cells were cultured in DMEM medium supplemented with 10% fetal bovine serum and 2 mM glutamine and transfected with FLAG-tagged ARMC5- or ARMC5 R315W-expressing plasmids by using Jet Prime Transfection Reagent (PolyPlus). The transfected cells were incubated at 37°C for 24 h, washed with PBS, pelleted, and snap-frozen until use. Affinity purifications were performed in four independent replicate experiments as described previously [93]. The Speedvac-dried protein extracts were re-solubilized in 10 μl of a 6 M urea buffer, reduced (45 mM DTT, 100 mM ammonium bicarbonate) for 30 min at 37°C, and alkylated (100 mM iodoacetamide, 100 mM ammonium bicarbonate) for 20 min at 24°C. Proteins were digested in 10 μl of trypsin solution (5 ng/μl of trypsin, Promega; 50 mM ammonium bicarbonate) at 37°C for 18 h. The digests were acidified with trifluoroacetic acid and cleaned by the Oasis MCX 96-well Elution Plate (Waters). Peptides were identified by LC–MS/MS using HPLC coupled to an Orbitrap Fusion mass spectrometer (Thermo Scientific) through a Nanospray Flex Ion Source. MS/MS raw data were searched against the human SwissProt database (updated on April 24th, 2019) and X-Tandem using ProHits software [94]. Spectral counts were transferred in Perseus (Version 1.6.1.3) [95]. Proteins quantified in three out of four experiments for either WT ARMC5 or ARMC5 R315W were kept for further analysis. Spectral counts reported as 0 by X-Tandem were replaced by a randomly generated spectral count value normally distributed with a mean and S.D. equal to those of the lowest 20% spectral count values from the LC–MS/MS analysis. Spectral counts were normalized by the spectral count of the bait (ARMC5) to allow comparison between different purifications. Proteins in the WT ARMC5 and ARMC5 R315W precipitates were compared to the FLAG empty vector control samples and were labeled as high-confidence interactors when their *p*-value was under 0.05. Their spectral count ratio was over 1.5. Statistically significant differences between proteins from the WT ARMC5 and ARMC5 R315W precipitates were determined using a two-tailed *t*-test. They were subsequently adjusted for multiple testing using a Benjamini-Hochberg-based test [96]. FDR of 5% was adjusted using a 0-correction factor of 0.1. The level of differential interaction was considered statistically significant when the FDR was < 0.05 and its average spectral count fold change between WT ARMC5 and ARMC5 R315W was >  ± 2.

### Immunoprecipitation and immunoblotting

Cells or tissues (i.e., human neuronal SK-N-SH cells or HEK293 cells transfected with human WT ARMC5- or mutant ARMC5-expressing plasmids, mouse NPCs, mouse e9.5 neural tubes or mouse intestine, and MEFs) were lysed in RIPA buffer (25 mM Tris, pH 7.6, 150 mM NaCl, 1% Nonidet P-40, 0.1% SDS) supplemented with protease inhibitors and phosphatase inhibitors (Roche Diagnostics). For immunoprecipitation, 0.5 mg of protein was incubated with mouse anti-HA mAb (clone HA-7, Sigma), mouse anti-ubiquitin mAb (clone F-11; Santa Cruz), mouse anti-POLR2A mAb (clone F-12; Santa Cruz Biotech), mouse anti-POLR2A mAb (clone 4H8; BioLegend) overnight, and then with protein G pre-conjugated agarose beads for additional 2 h at 4 °C with rotary agitation. The beads were washed with lysis buffer four times and eluted in SDS-loading buffer. For immunoblotting, the lysates were resolved by 6 to 12% SDS-PAGE and transferred to nitrocellulose membranes. The membranes were blocked with 5% (w/v) milk in TBST (Tris-Buffered Saline, 0.05% Tween 20) and incubated with first Abs overnight at 4 °C, followed by HRP (horse radish peroxidase)-conjugated secondary Abs for 1 h at room temperature. In some cases, HPR-conjugated first Abs without a second Ab were used. The signal was revealed by the Western Lightening pro-ECL (PerkinElmer) or SuperSignal West Pico Chemiluminescent Substrate (Thermo Scientific) and detected with X-ray film.

Following first Abs or HRP-conjugated first Abs were used for blotting. HRP-conjugated anti-HA- mAb (clone 6E2Cell Signaling Technology), mouse anti-FLAG mAb (clone M2; Sigma-Aldrich), mouse anti-CUL3 mAb (clone G-8; Santa Cruz), mouse anti-POLR2A mAb (clone 4H8; BioLegend), mouse anti-POLR2A mAb (clone 8WG16; BioLegend), rabbit anti-phospho-POLR2A-S2 mAb (clone E1Z3G; Cell Signaling Technology), rabbit anti-phospho-POLR2A-S5 mAb (clone D9N51; Cell Signaling Technology), mouse anti-ubiquitin mAb (clone F-11; Santa Cruz Biotech), mouse anti-FOLH1 mAb (clone OTI3H5; Novus Biologicals), rabbit anti-β-actin Ab (Cell Signaling Technology), rabbit anti-α-actinin mAb (clone D6F6; Cell Signaling Technology) or mouse anti-K48-ubiquitin mAb (clone Apu2; Millipore/Sigma), mouse anti-POLR2A mAb (clone F-12; Santa Cruz), mouse anti-POLR2B mAb (clone E-12; Santa Cruz), rabbit anti-POLR2C mAb (clone EPR13294(B); Abcam), rabbit anti-POLR2D Ab (Abcam), mouse anti-POLR2E mAb (clone B-5; Santa Cruz), mouse anti-POLR2F mAb (clone E-8; Santa Cruz), mouse anti-POLR2G mAb (clone C-2; Santa Cruz), mouse anti-POLR2H mAb (clone B8-1; Santa Cruz), mouse anti-POLR2I mAb (clone F-11; Santa Cruz), mouse anti-POLR2J mAb (clone G-2; Santa Cruz), rabbit anti-POLR2K Ab (ThermoFisher), rabbit anti-POLR2L Ab (ThermoFisher), rabbit anti-β-actin rabbit Ab (Cell Signaling Technology), and rabbit anti-α-actinin mAb (clone D6F6; Cell Signaling Technology).

### Construction of a 3D model of the E3 and Pol II complex

The structure of ARMC5 was obtained from AlphaFold2 [64, 65]. The structures of Pol II and other components of the E3 were extracted from the Protein Data Bank (PDB). ChimeraX [97] was used to construct the 3D model of the complex. The components in the complex were positioned according to the information derived from our previous deletion studies [45] and PDB.

### RNA-seq

The total RNA of three pairs of biological replicates of KO and WT NPCs was extracted by the Rneasy kit (Qiagen). The total RNA was quantified using a NanoDrop Spectrophotometer ND-1000 (NanoDrop Technologies), and its integrity was assessed on a 2100 Bioanalyzer (Agilent Technologies). rRNA was depleted from 250 ng of total RNA using QIAseq FastSelect (Human 96rxns; Qiagen). Library construction, quantification, normalization, and sequencing were conducted as described elsewhere [45]. Data processing such as read trimming, clipping and alignment, and differential expression analysis were carried out as described previously [45]. The mapping was conducted at the transcript level.

Out of the original transcripts, 47,059 transcripts were left after filtering. It is to be noted that one gene could have several different transcript isoforms due to alternative splicing or the use of varying initiation sites.

Each gene was tested for differential expression between WT and KO NPCs with an EdgeR LRT test. Due to the concern that the augmented Pol II pool caused by ARMC5 deletion might generally affect all the gene transcription in the KO NPCs, the raw counts of each transcript were normalized by the ratio between the log_2_ counts per million reads of *Rn7sk* of a particular sample to the average *Rn7sk* log_2_ counts per million reads of across all samples. *Rn7sk* was transcribed by Pol III and was thus independent of the putative influence of the Pol II pool size. This normalization was used instead of using edgeR::calcNormFactors, which uses a trimmed mean of M values normalization by default.

The heatmap was constructed using R pheatmap. The volcano plots and bar plots were produced using R v3.6.3. ggplot2. Based on a threshold for gene-level significance of 5% FDR, GO analysis of the RNA-seq data was performed using the Cytoscape v3.7.2 application ClueGO v2.5.6. The Uniprot Gene Ontology Annotations were used to classify the GO terms.

### POLR2A ChIP-Seq

Three biological replicate pairs of KO and WT NPCs were washed with ice-cold PBS twice and re-suspended in 1 ml PBS. They were crosslinked by adding 66.7 µl of 16% formaldehyde (1% final) at room temperature for 15 min. The reaction was quenched with 107 µl of 1.25 M glycine (0.125 M final) at room temperature for another 10 min in rotating tubes. The samples were centrifuged, and the pellets were washed twice with ice-cold PBS. The crosslinked pellets were suspended in 300 µl swelling buffer (25 mM HEPES, pH 7.5, 1.5 mM MgCl_2_, and 10 mM KCl, 0.1% NP-40) and incubated on ice for 20 min to release nuclei. The nuclei were harvested by centrifugation, re-suspended in 200 µl ChIP sonication buffer (50 mM HEPES, pH 7.5, 140 mM NaCl, 1 mM EDTA, 0.1% Na-deoxycholate, 1% Triton X-100, 0.1% SDS), and incubated on ice for 20 min. The nuclei were sonicated with a probe-based sonicator (model FB120, CL-18 probe; Fisher Scientific) at a 25% amplitude setting. The sonication was conducted using 30-s pulses at 30-s intervals for a total of 5 min. The sonicated nuclei representing chromatin were harvested by centrifugation and were ready for immunoprecipitation.

To quantify chromatin and assess the degree of its fragmentation, we treated 5% of the sonicated nuclei (10 µl/sample) with 10 µg of RNase A for 15 min at 37 °C, followed by 20 µg of proteinase K for 30 min at 65 °C. They were quickly de-crosslinked for 5 min at 95 °C. DNA was extracted with the QIAquick PCR Purification Kit (Qiagen). DNA concentration was determined with a Nanodrop 1000 Fluorospectrometer. DNA fragment sizes were confirmed to be mainly at the 100–800-bp range according to electrophoresis.

For immunoprecipitation, an equal amount (based on their DNA measurements) of sonicated chromatin of different samples was reacted with mouse anti-POLR2A N-terminal domain mAb (clone D8L4Y, Cell Signaling Technology) (1:100) at 4 °C overnight, followed by 40 µl magnetic protein G beads (Bio-Rad) for another 2 h at 4 °C. The beads were rinsed once with sonication buffer, once with wash buffer A (50 mM HEPES, pH 7.5, 500 mM NaCl, 1 mM EDTA, 0.1% Na-deoxycholate, 1% Triton X-100, 0.1% SDS), once with wash buffer B (20 mM Tris, pH 8.0, 250 mM LiCl, 1 mM EDTA, 0.5% NP-40, 0.5% Na-deoxycholate) and then twice with TE buffer (10 mM Tris, pH 8.0, 1 mM EDTA). The chromatin was eluted with elution buffer (50 mM Tris, pH 8.0, 10 mM EDTA, 1% SDS) at 65 °C for 10 min. The immunoprecipitated chromatins were de-crosslinked at 65 °C overnight with NaCl adjusted to 540 mM. The chromatins were then treated with 10 µg RNase A/sample at 37 °C for 1 h, followed by 40 µg proteinase K/sample for 2 h at 45 °C. DNA of the samples was purified with QIAquick PCR Purification kit (Qiagen) and quantified by the Bioanalyzer (Agilent).

Libraries were prepared robotically with 2–10 ng of fragmented DNA ranging from 100 to 300 bp in length, using the NEBNext Ultra II DNA Library Prep Kit for Illumina (New England BioLabs), as per the manufacturer’s recommendations. Adapters and PCR primers were purchased from Integrated DNA Technologies. Size selection was carried out using SparQ beads (Qiagen) prior to PCR amplification (12 cycles). Libraries were quantified using the Kapa Illumina GA with Revised Primers-SYBR Fast Universal kit (Kapa Biosystems). Average fragment sizes were determined using a LabChip GX (PerkinElmer) instrument.

The library construction and sequencing were the same as described elsewhere [45]. Downstream data processing, such as ChIP-seq read trimming, alignment, peak calling, and annotation, was performed as described before [45].

To assess differences in Pol II occupancy patterns between WT and KO samples, we obtained ChIP-seq read counts within the following genomic regions using HOMER: the promoter region (from TSS (transcription start site) − 400 bp to TSS + 100 bp), the gene body (from TSS + 100 bp to TES (transcription end site) − 100 bp), the TES region (from TES − 100 bp to TES + 2000 bp; also called the downstream region), the 5′ untranslated region (5′UTR), introns, 3′UTR, enhancers (from TSS − 5000 bp to TSS − 400 bp), the region from − 10,000 bp to TSS, the region from TSS to + 10,000 bp, and the intergenic region. Since the POLR2A levels in the KO tissues were elevated, we speculated that there would be more Pol II association with the genes, hence higher POLR2A ChIP signal in the KO promoter regions than in the WT counterparts. Therefore, genes that lacked POLR2A ChIP-seq signal in the KO tissues were filtered out, as these genes were believed to have no signals in WT tissues either. Raw counts were normalized using edgeR’s trimmed mean of M algorithm [98] and were then transformed to log_2_ counts per million using the Voom function implemented in the Limma R package [99].

To construct the global metagene Pol II-binding profile, normalized read counts (fragments per kilobase of transcript per million mapped reads of the full gene length plus 2000-bp flanks (TSS − 2000 bp to TES + 2,000 bp) were obtained from all the genes that passed the filtering. Both flanks were divided into 20 equal-sized bins of 100 bp each. The gene bodies were scaled to 60 bins for the full gene length. FPKM was calculated from BAM input files using ngs.plot [100] with the following parameters: -G mm10 -R genebody -D ensembl -FL 200 -BOX 0 -SE 1 -VLN 0 -LWD 2 -WD 9. These global metagene Pol II-binding profiles were only for visualization of differences in Pol II density, and inferential statistics were not conducted as per custom.

The peak count versus distance (− 10 kb to + 10 kb from TSS) profile was generated from 51 equal-sized bins of 400 bp for this region of all the genes that passed filtering.

Differential Pol II peak density analysis in WT and KO tissues was conducted as described before [45]. We calculated the pausing index for each gene by computing the ratio of Pol II signal density in the promoter region (from TSS − 400 bp to TSS + 100 bp) to signal density within the gene body (from TSS + 100 bp to TES + 2 kb) as follows:

$$Pausing\;index\;(PI)\;=\;\frac{Promoter\;region\;FPKM/L1}{Genebody\;FPKM/L2}$$

L1 is the length of the promoter region (always 500 bp), and L2 is the length of the gene body (variable).

Genome browser tracks were created with the HOMER makeUCSCfile command and bedGraphToBigWig utility from UCSC. Tracks were normalized so that each value represented the read count per base pair per 10 million reads. UCSC Genome Browser (http://genome.ucsc.edu/) was implemented for track visualization.

### Nuclear run-on assay

Nuclear run-on assays were carried out according to a step-by-step protocol by Roberts et al. [101]. Briefly, nuclei from 4 × 10^6^ KO or WT NPCs were collected and transcribed with Br-UTP and other NTPs. Nuclear RNA was extracted using the MEGAclear transcription clean-up kit (Life Technologies), and genomic DNA contamination was removed using the TURBO DNA-free kit (Life Technologies). Br-UTP-incorporated nascent transcripts were precipitated with anti-BrdU mAb (Santa Cruz Biotechnology), extracted, and reverse-transcribed using a high-capacity cDNA reverse transcription kit (Invitrogen). qPCR was performed to quantify the nascent mRNA. To empirically determine the sensitivity of detecting nascent transcripts and the purity of Br-UTP-incorporated nascent transcripts over UTP-containing transcripts, before reverse transcription of the nuclear run-on reactions, we spiked the test samples with separately prepared control bacterial oligonucleotides with or without incorporated Br-UTP at known concentrations.

### MM study cohort

A total of 511 subjects were selected for whole-exome sequencing from an MM study cohort enrolled in *spina bifida* clinics in five locations in North America between 1997 and 2010 [102]. All the subjects were consented to and enrolled in accordance with an institutional Internal Review Board at the University of Texas Health Science Center at Houston. In total, samples of 257 MM subjects of European descent, comprising 140 females and 117 males, and 254 Mexican–American MM subjects comprising 134 females and 120 males, were sequenced. Three hundred and sixty-five of the study subjects (over 70%) were born before January 1998, when the North American countries mandated folic acid fortification of food crops. Sixty subjects were born in 1998, and 86 after 1998. Blood samples were collected from the subjects, and genomic DNA was extracted for the study.

### Exome sequencing and variant annotation

Exome library probes were made from an in-house design based on TargetSeq (Invitrogen) with the addition of splice sites, UTRs, small non-coding RNAs (e.g., microRNAs), and a selection of miRNA binding sites, and 200-bp promoter regions. High-quality genomic DNA samples were processed using the exome library probes, and the captured DNA products were sequenced following the manufacturer’s standard protocol for multiplexed sequencing using the P1 chip on the Ion Proton platform (Invitrogen). Quality of sequencing was maintained at 40–60 million reads/sample with read length between 120 and 150 bases, and over 75% of reads were on target for all successfully sequenced samples. Other quality controls were implemented to map around 45,000–60,000 single-nucleotide variants (SNV) per sample with ~ 50% heterozygote variants and the transition/transversion ratio around 2.5. Samples that failed to meet the above quality criteria were repeated or substituted by another subject’s DNA.

Sequence data passed the above variant- and sample-quality filters were processed to call variants using Genome Analysis Toolkit HaplotypeCaller version 3.x, following best-practice guidelines. Briefly, only variants designated a “PASS” by Variant Quality Score Recalibration and having mapping quality score > 20, or inbreeding coefficient <  − 0.3, were retained for further analysis. Individual sample filters were used to ensure only high-fidelity variants with an alternate allele depth > 25%, a read depth > 10, and a genotype quality score > 20. The allele count, allele number, and allele frequency were recalculated for individual ethnicities after the filtering processes. Filtered high-quality SNVs were annotated using the non-synonymous SNV functional predictions database [103] with an in-house Python script for all current functional prediction information publicly available. Further analyses were focused on single SNVs leading to stop-gained, stop-lost, non-synonymous, splice donor, and acceptor site changes in canonical transcripts.

### Novel functional deleterious SNV analysis

To analyze SNVs, we referred to AFs of variants observed in the non-Finnish European and Ad Mixed American populations of the genome aggregation database (gnomAD) Exome Controls [104]. Variants not observed in the non-Finnish European or Ad Mixed American gnomAD Exome Controls or having ethnic allele frequency = 0 were defined as novel SNVs (nSNVs). Datasets of non-Finnish Europeans or Ad Mixed Americans in gnomAD Exome Controls were downloaded for extracting alternate allele counts and total allele counts of all variants identified in MM subjects for comparison using the sample filters described previously [105]. For novel variants identified in subjects but not in gnomAD, we further verified that the loci were sequenced in gnomAD with ≥ 30X coverage, and the corresponding variants were absent. Loci with < 30X coverage were considered poor in quality and were discarded. These loci in the gnomAD controls were interpreted as having the reference alleles only, and the alternate allele frequency was considered to be zero.

nSNVs identified in the MM subjects were further verified by Sanger sequencing. PCR primers franking 200 to 300 bases from the variants were designed to amplify the variant-containing loci from the MM subjects. The amplified loci were then sequenced.

Variants with allele frequency in non-Finnish Europeans or Ad Mixed Americans less than 0.01 were defined as rare, while allele frequency ≥ 0.01 was defined as common. Combined Annotation Dependent Depletion [71] (C-score) of variants was used as a model to predict deleteriousness. C-score is the -10 × log % rank of deleteriousness. A variant with a C-score of 13.01 is among the top 5% of most deleterious variants, and a variant with a C-score of 20 is among the top 1%.

For alternate allele counts between the MM subjects and gnomAD Exome Controls, odds ratios were calculated, and the Fisher tests were performed.

Analysis of variants within the *ARMC5* transcript (NM_001288767) for linkage disequilibrium (LD) was carried out using Idlink [106].

### Construction of mutant ARMC5-expressing plasmids

Plasmids expressing human ARMC5 mutants ARMC5(P33S)-FLAG, ARMC5(R334C)-FLAG, ARMC5(R406Q)-FLAG, ARMC5(G422S)-FLAG, ARMC5(P559L)-FLAG, and ARMC5(R793Q)-FLAG were generated by mutating WT ARMC5-FLAG-expressing plasmid (EX-H0661-M11, GeneCopoeia, Rockville, US), using KOD Xtreme Hot Start DNA polymerase (71,975, Millipore-Sigma, US) and the Q5 Site-Directed Mutagenesis Kit (E0554S, New England Biolabs, ON, Canada).

### Supplementary Information

**Additional file 1: Figure S1.** Similar Rn7sk expression in WT and KO NPCs according to RNA-seq.  **Table S1.** Detailed parameters of differentially expressed transcripts in WT versus KO NPCs according to RNA-seq.  **Table S2.** GO analysis of significantly dysregulated genes in terms of biological process. **Table S3.** Genes with highly different Pol II peak density (FDR<0.1) between WT and KO NPCs. **Table S4.** RT-qPCR primer sequences.  Uncropped blots. 

**Additional file 2.**  Review history.

## Data Availability

The mouse RNA-seq and ChIP-seq datasets have been deposited to the Gene Expression Omnibus of NCBI (accession numbers GSE169350 and GSE169582, respectively) [53, 70]. Microscopy images have been submitted to Figshare [46, 49, 54]. Mass spectrometry data are available via proteomeXchage (accession numbers: PXD047533 and PXD047572) [72]. Unique propagatable materials used in this study are available to qualified researchers upon request.
