# Supplementary material for: ARMC5 controls the degradation of most Pol II subunits, and ARMC5 mutation increases neural tube defect risks in mice and humans
Source: Genome Biol. 2024 Jan 15;25:19. doi: 10.1186/s13059-023-03147-w (PMC10789052; doi:10.1186/s13059-023-03147-w)

**Additional file 1**

**Figure S1.** **Similar Rn7sk expression in WT and KO NPCs according to RNA-seq**

*Rn7sk* levels in WT and KO NPCs were determined by RNA-seq. Log_2_CPM of *Rn7sk* of 3 WT and 3KO NPC samples are presented. FDR of the differences between the WT and KO samples is not significant.


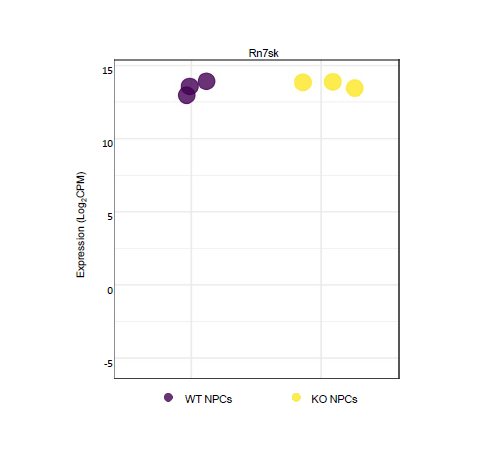

Supplement: Supplementary file 1 — Additional file 1: Figure S1. Similar Rn7sk expression in WT and KO NPCs according to RNA-seq. Table S1. Detailed parameters of differentially expressed transcripts in WT versus KO NPCs according to RNA-seq. Table S2. GO analysis of significantly dysregulated genes in terms of biological process. Table S3. Genes with highly different Pol II peak density (FDR<0.1) between WT and KO NPCs. Table S4. RT-qPCR primer sequences. Uncropped blots. [file 13059_2023_3147_MOESM1_ESM.zip › Additional file 1 Figure S1 2023-12-7.docx]
