# Supplementary material for: ARMC5 controls the degradation of most Pol II subunits, and ARMC5 mutation increases neural tube defect risks in mice and humans
Source: Genome Biol. 2024 Jan 15;25:19. doi: 10.1186/s13059-023-03147-w (PMC10789052; doi:10.1186/s13059-023-03147-w)
Supplement: Supplementary file 1 — Additional file 1: Figure S1. Similar Rn7sk expression in WT and KO NPCs according to RNA-seq. Table S1. Detailed parameters of differentially expressed transcripts in WT versus KO NPCs according to RNA-seq. Table S2. GO analysis of significantly dysregulated genes in terms of biological process. Table S3. Genes with highly different Pol II peak density (FDR<0.1) between WT and KO NPCs. Table S4. RT-qPCR primer sequences. Uncropped blots. [file 13059_2023_3147_MOESM1_ESM.zip › Additional file 6 uncropped blots 2023-12-7.pptx]

## Slide 1
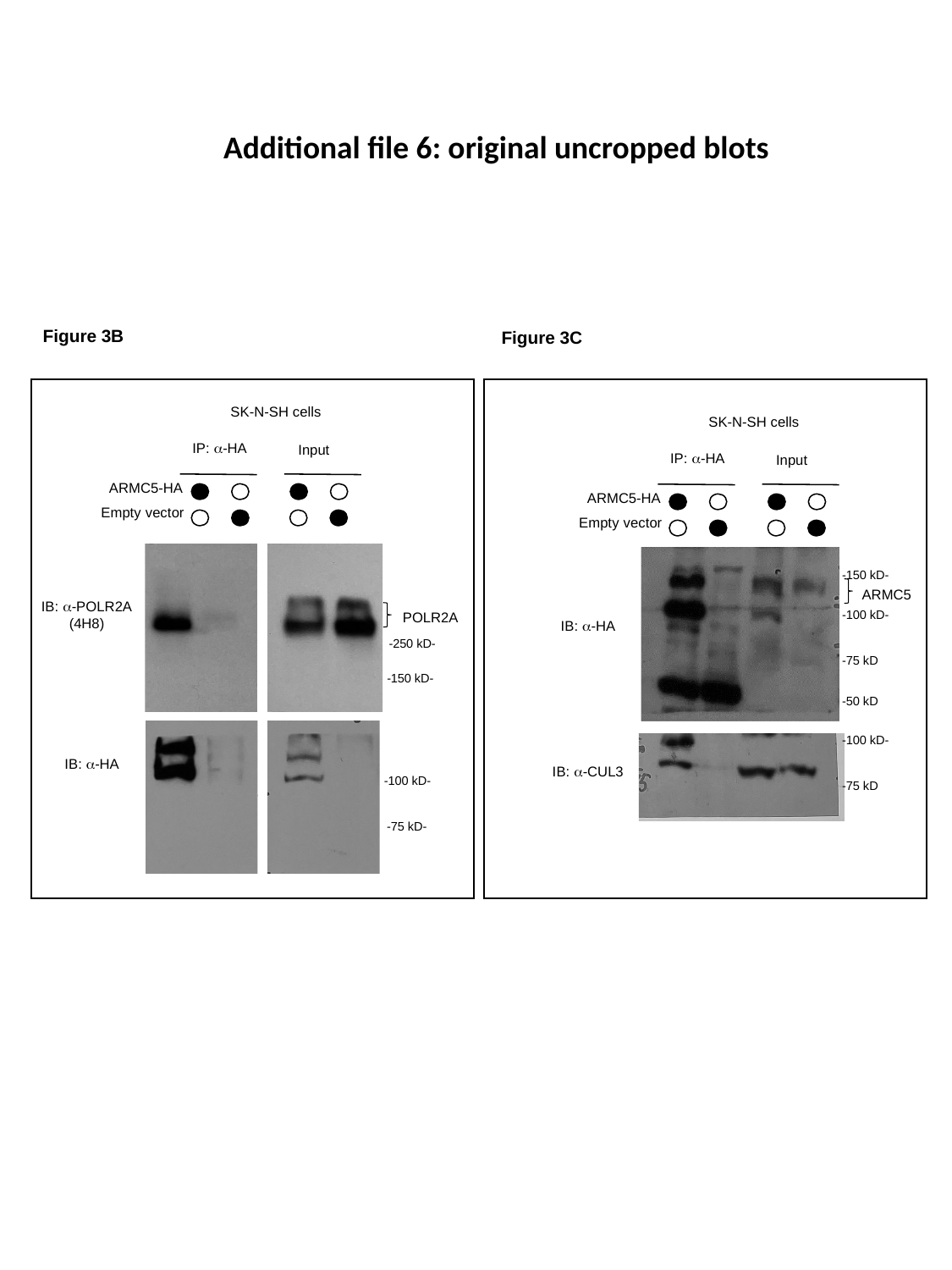

Additional file 6: original uncropped blots
Figure 3B
Figure 3C
SK-N-SH cells
SK-N-SH cells
IP: a-HA
Input
ARMC5-HA
Empty vector
IP: a-HA
Input
ARMC5-HA
Empty vector
-150 kD-
ARMC5
IB: a-POLR2A
(4H8)
-100 kD-
POLR2A
IB: a-HA
-250 kD-
-75 kD
-150 kD-
-50 kD
-100 kD-
IB: a-HA
IB: a-CUL3
-100 kD-
-75 kD
-75 kD-

## Slide 2
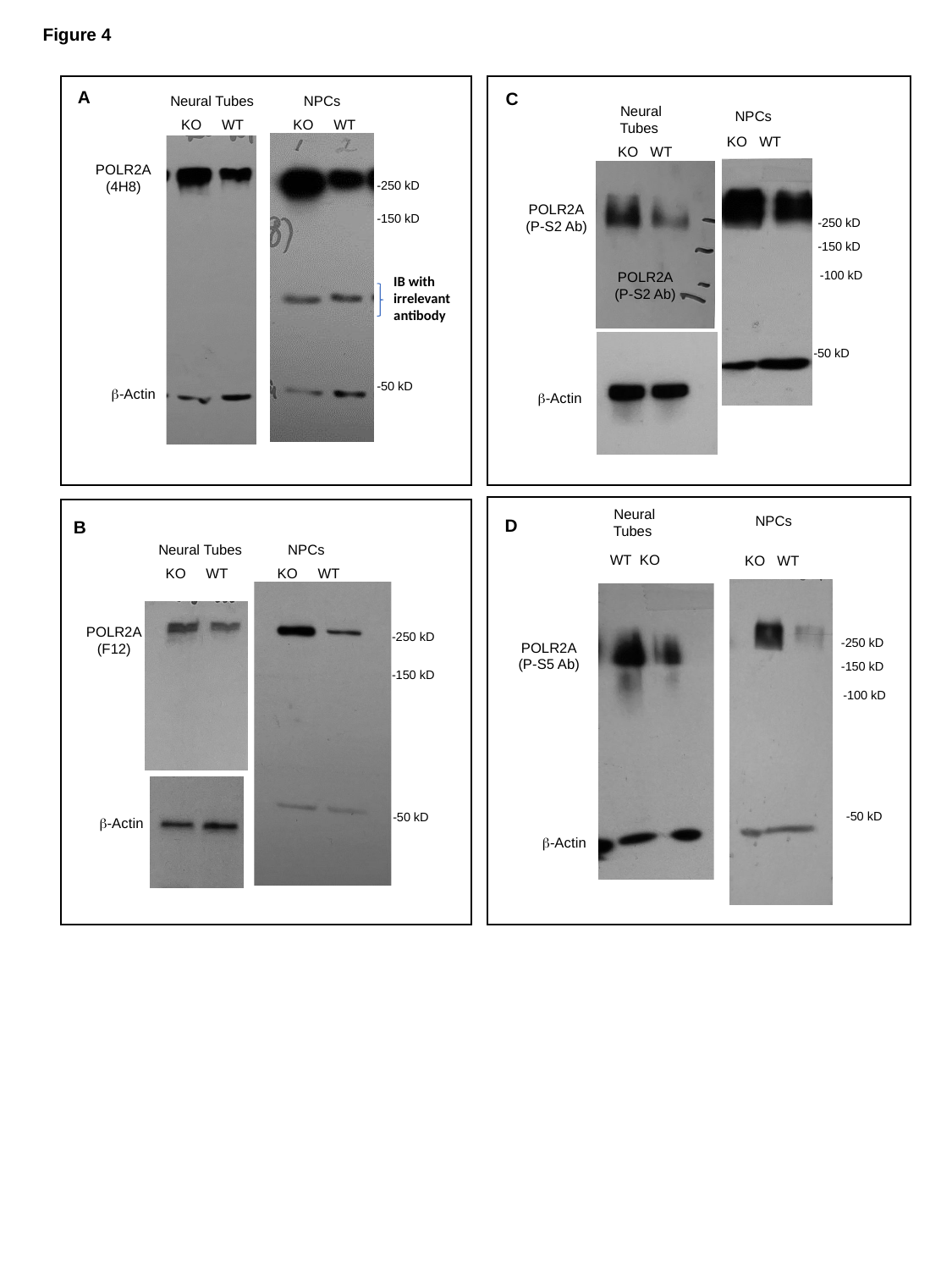

Figure 4
A
C
Neural Tubes
NPCs
KO WT
KO WT
Neural Tubes
NPCs
KO WT
KO WT
POLR2A
(4H8)
-250 kD
-150 kD
-50 kD
POLR2A
(P-S2 Ab)
-250 kD
-150 kD
-100 kD
POLR2A (P-S2 Ab)
IB with irrelevant antibody
-50 kD
b-Actin
b-Actin
Neural Tubes
NPCs
WT KO
KO WT
D
B
Neural Tubes
NPCs
KO WT
KO WT
POLR2A
(F12)
-250 kD
-150 kD
-50 kD
-250 kD
-150 kD
-100 kD
POLR2A
(P-S5 Ab)
-50 kD
b-Actin
b-Actin

## Slide 3
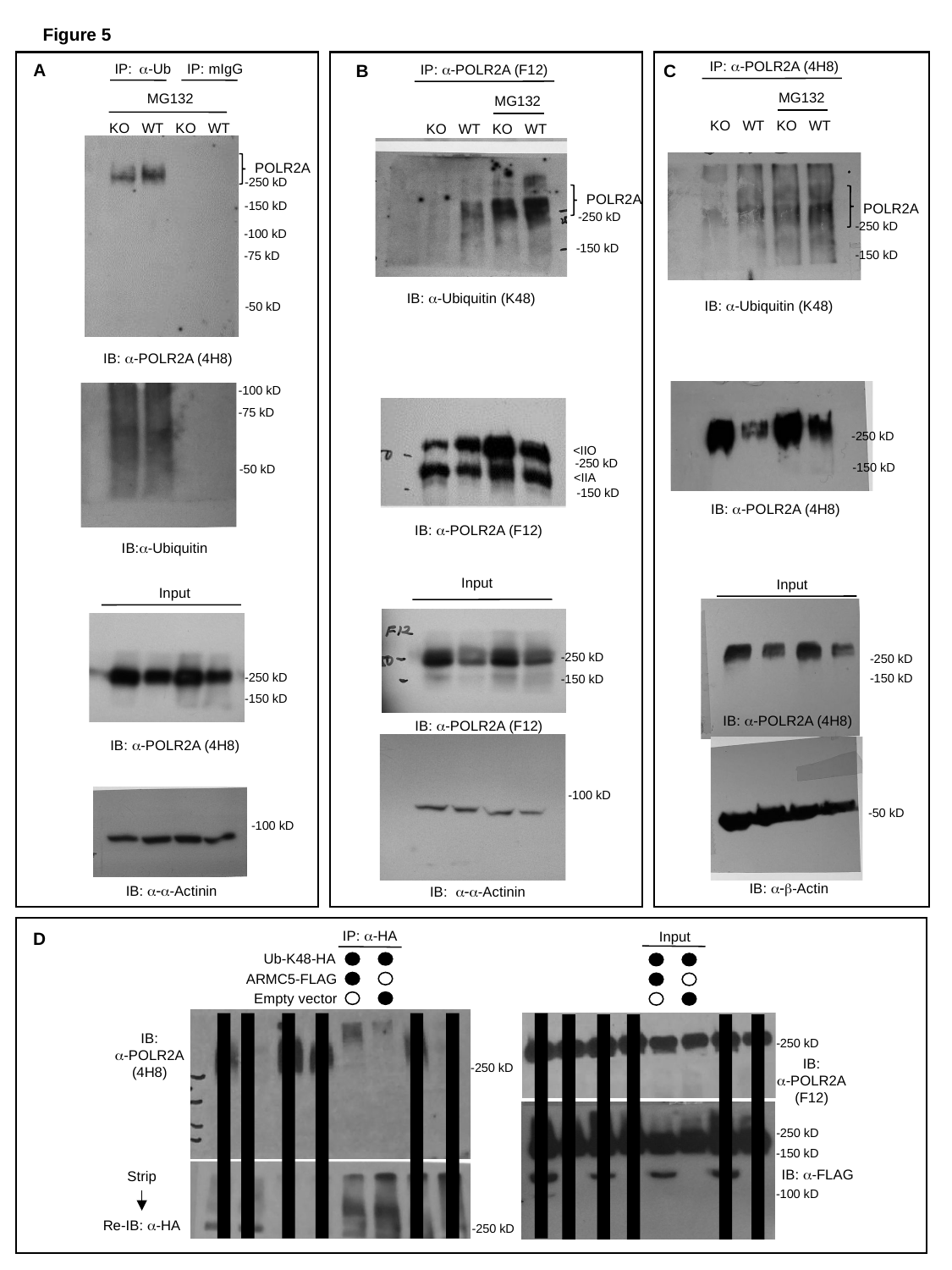

Figure 5
IP: a-POLR2A (4H8)
A
B
C
IP: a-Ub IP: mIgG
IP: a-POLR2A (F12)
MG132
KO WT KO WT
MG132
KO WT KO WT
MG132
KO WT KO WT
POLR2A
-250 kD
-150 kD
POLR2A
POLR2A
-250 kD
-150 kD
-250 kD
-150 kD
-100 kD
-75 kD
-50 kD
IB: a-Ubiquitin (K48)
IB: a-Ubiquitin (K48)
IB: a-POLR2A (4H8)
-100 kD
-75 kD
-50 kD
-250 kD
-150 kD
<IIO
-250 kD
-150 kD
<IIA
IB: a-POLR2A (4H8)
IB: a-POLR2A (F12)
IB:a-Ubiquitin
Input
Input
Input
-250 kD
-150 kD
-250 kD
-150 kD
-250 kD
-150 kD
IB: a-POLR2A (4H8)
IB: a-POLR2A (F12)
IB: a-POLR2A (4H8)
-100 kD
-50 kD
-100 kD
IB: a-b-Actin
IB: a-a-Actinin
IB: a-a-Actinin
IP: a-HA
Input
Ub-K48-HA
ARMC5-FLAG
Empty vector
IB:
a-POLR2A
(4H8)
-250 kD
IB:
a-POLR2A
(F12)
-250 kD
-250 kD
-150 kD
-100 kD
IB: a-FLAG
Strip
Re-IB: a-HA
-250 kD
D

## Slide 4
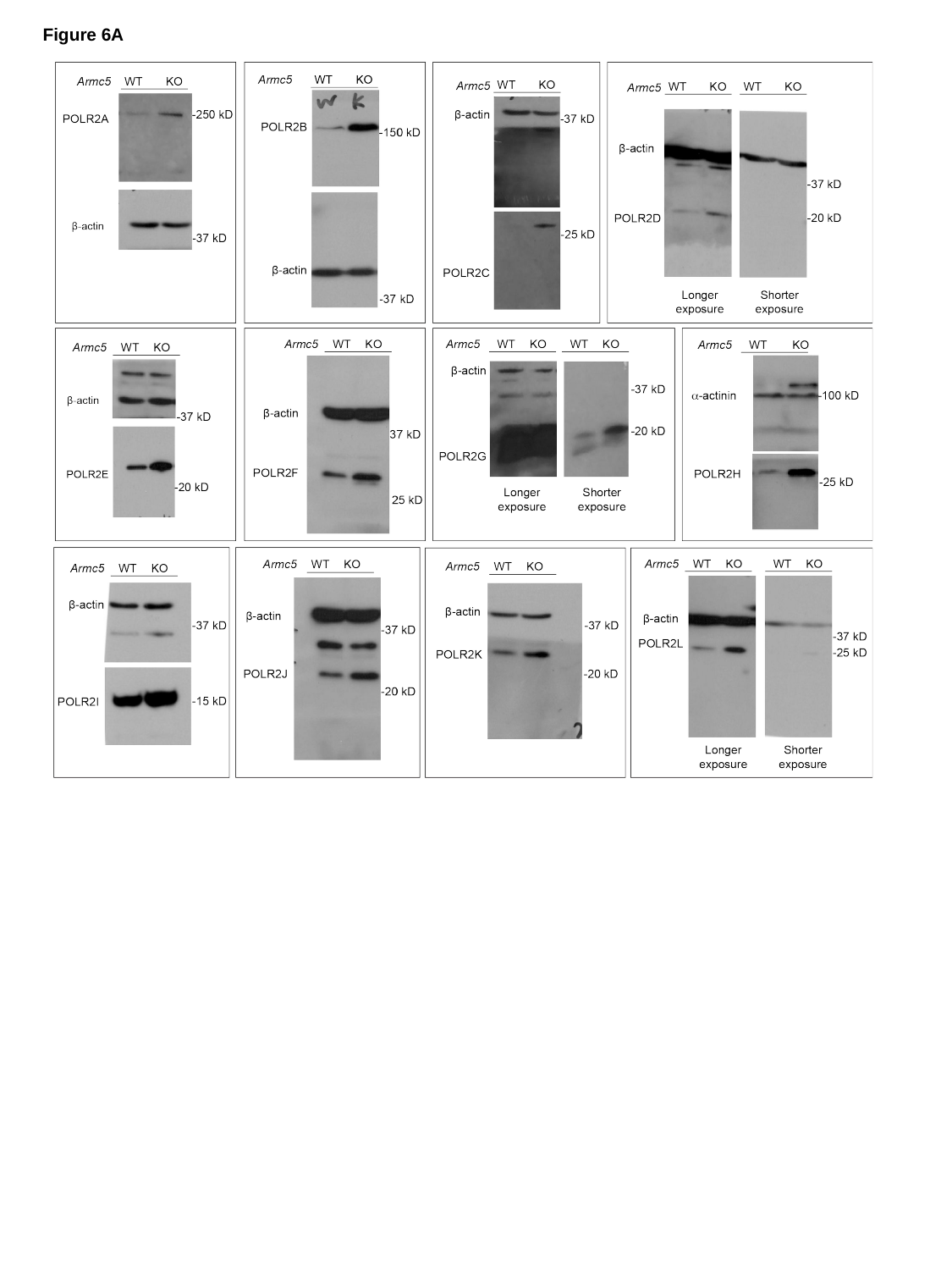

Figure 6A

## Slide 5
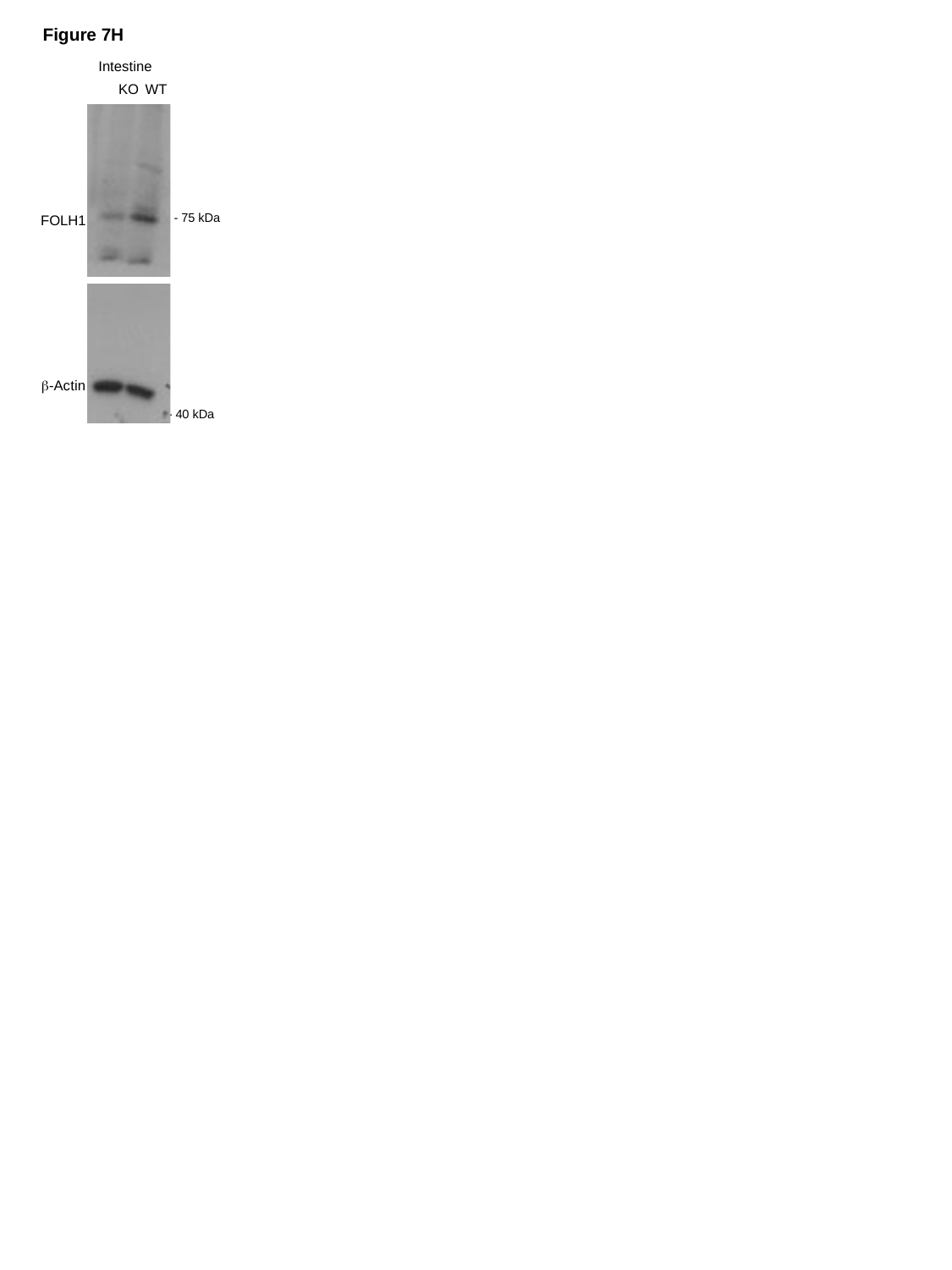

Figure 7H
Intestine
WT
KO
- 75 kDa
FOLH1
b-Actin
- 40 kDa

## Slide 6
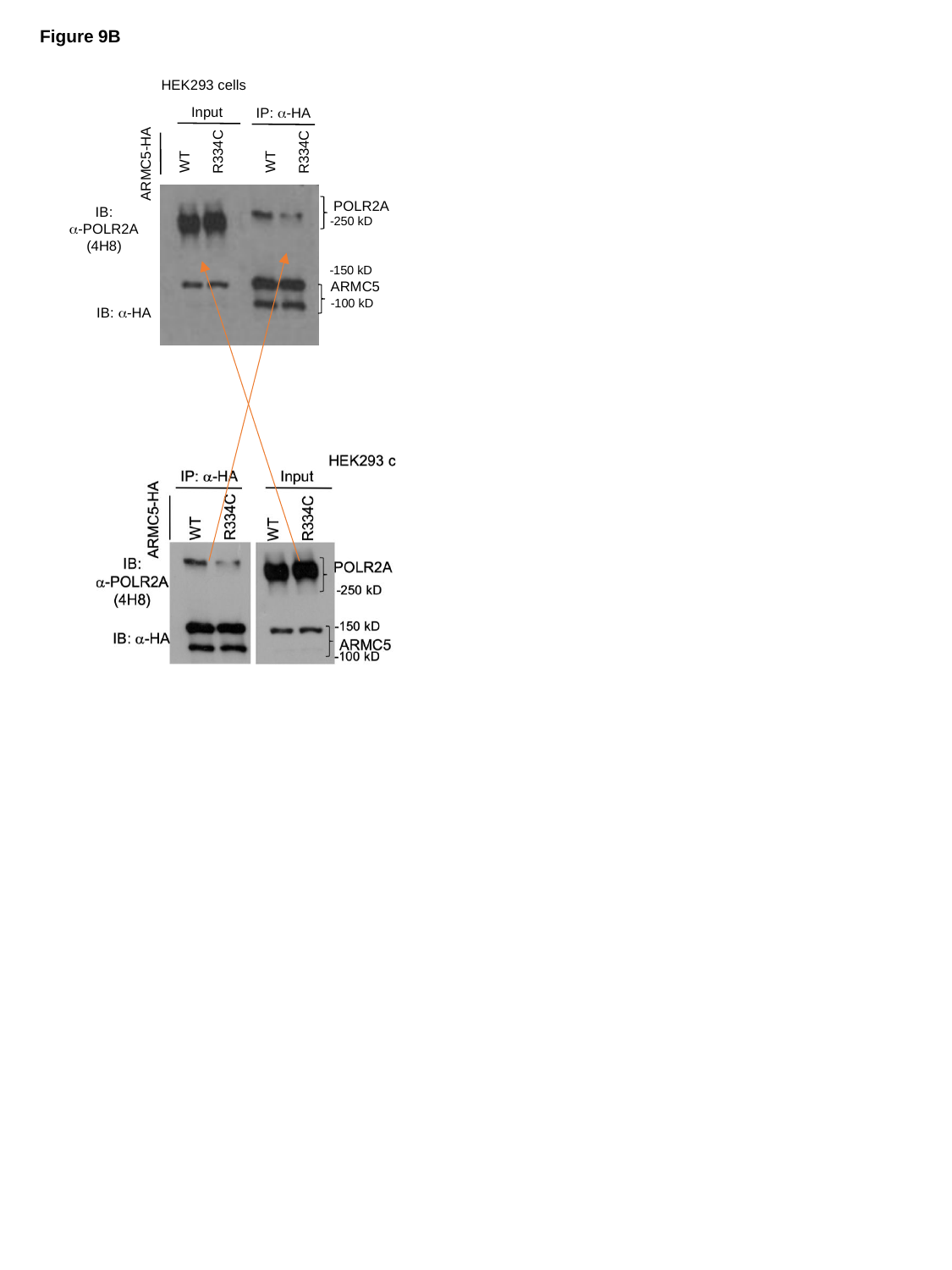

Figure 9B
HEK293 cells
Input
IP: a-HA
R334C
R334C
ARMC5-HA
WT
WT
POLR2A
IB:
a-POLR2A
(4H8)
-250 kD
-150 kD
ARMC5
-100 kD
IB: a-HA

## Slide 7
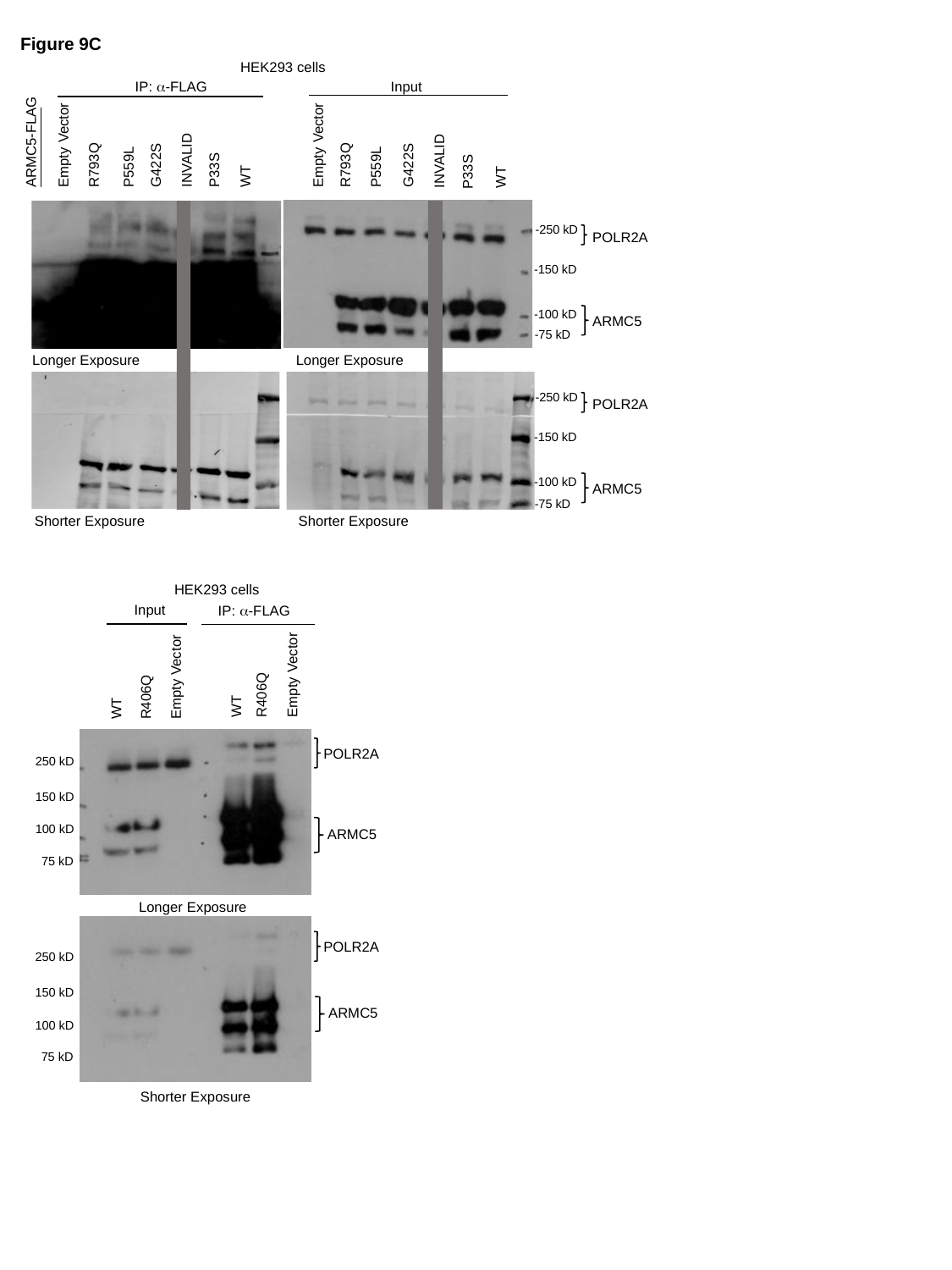

Figure 9C
HEK293 cells
IP: a-FLAG
Input
Empty Vector
R793Q
P559L
G422S
INVALID
P33S
WT
Empty Vector
R793Q
P559L
G422S
P33S
WT
ARMC5-FLAG
INVALID
-250 kD
POLR2A
-150 kD
-100 kD
ARMC5
-75 kD
Longer Exposure
Longer Exposure
-250 kD
POLR2A
-150 kD
-100 kD
ARMC5
-75 kD
Shorter Exposure
Shorter Exposure
HEK293 cells
Input
IP: a-FLAG
Empty Vector
R406Q
WT
Empty Vector
R406Q
WT
POLR2A
250 kD
150 kD
100 kD
ARMC5
75 kD
Longer Exposure
POLR2A
250 kD
150 kD
ARMC5
100 kD
75 kD
Shorter Exposure

## Slide 8
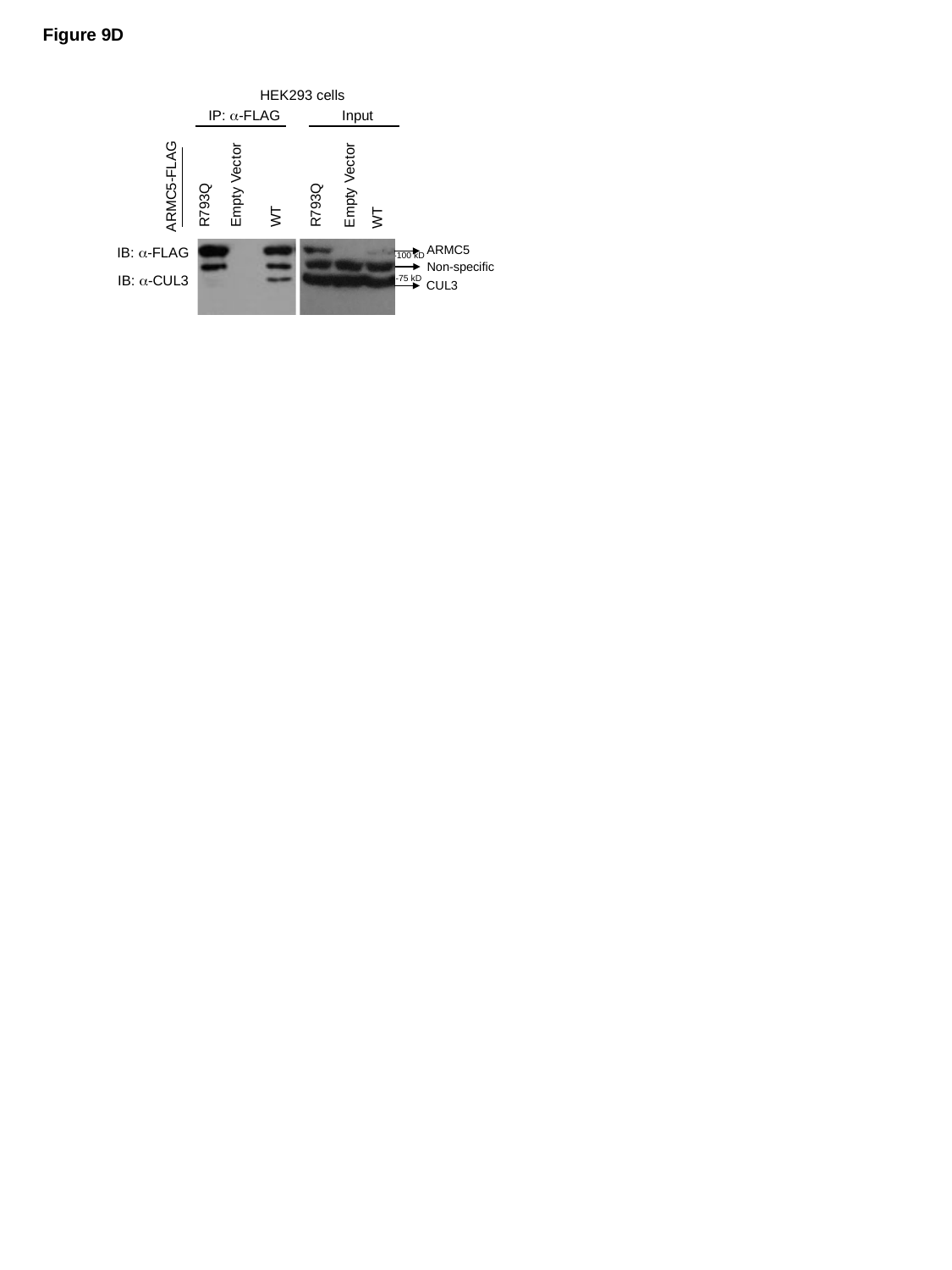

Figure 9D
HEK293 cells
IP: a-FLAG
Input
Empty Vector
ARMC5-FLAG
Empty Vector
R793Q
R793Q
WT
WT
ARMC5
IB: a-FLAG
-100 kD
Non-specific
IB: a-CUL3
-75 kD
CUL3

## Slide 9
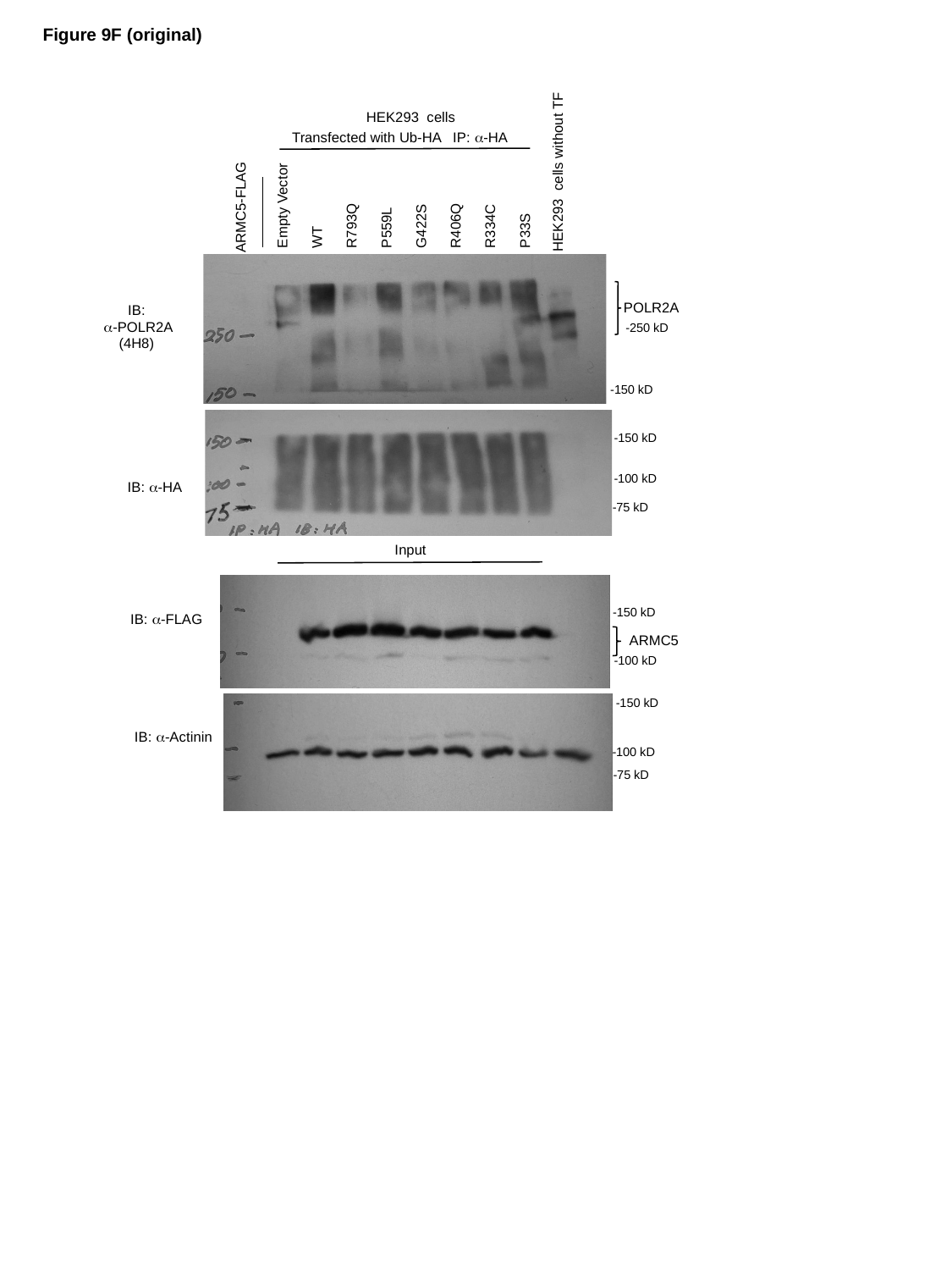

Figure 9F (original)
HEK293 cells
Transfected with Ub-HA IP: a-HA
Empty Vector
G422S
R406Q
P559L
R793Q
P33S
R334C
WT
HEK293 cells without TF
ARMC5-FLAG
POLR2A
IB:
 a-POLR2A
(4H8)
-250 kD
-150 kD
-150 kD
-100 kD
IB: a-HA
-75 kD
Input
-150 kD
IB: a-FLAG
ARMC5
-100 kD
-150 kD
IB: a-Actinin
-100 kD
-75 kD
