# Supplementary material for: ARMC5 controls the degradation of most Pol II subunits, and ARMC5 mutation increases neural tube defect risks in mice and humans
Source: Genome Biol. 2024 Jan 15;25:19. doi: 10.1186/s13059-023-03147-w (PMC10789052; doi:10.1186/s13059-023-03147-w)
Supplement: Supplementary file 2 — Additional file 2. Review history. [file 13059_2023_3147_MOESM2_ESM.docx]

This article was previously reviewed at another journal. We have seen the reviewer reports and the authors’ responses to them, and know the identity of one of the reviewers. We had an independent reviewer assess the authors responses to the last round of reviews.

**Reviewer 1**

In this study, Luo et al. show three main findings: (1) altered ARMC5 is linked to neural tube defects (NTDs), (2) ARMC5 recruits the Ubiquitin ligase machinery to RNA polymerase II (Pol II) for its degradation, affecting the entire Poll II complex levels, and (3) disrupted regulation of Pol II by ARMC5 may lead to NTDs. Finding (1) is based on mouse as well as human genetics. Findings (2) and (3) are based on biochemistry experiments in Armc5 KO mouse tissue and cells, as well as in HEK cells. I found that findings (1) and (2) are well supported by the data but finding (3) is not completely supported, although the current data does make a good case that it is. Moreover, the authors do concede that finding (3) will require future studies to flesh out the underlying mechanism and the cause-and-effect relationship. To me, overall, the data is strong but the writing of this manuscript has two facets, it can be either very good (the biochemistry and human data part) or on the contrary really hard to read (the mouse phenotyping portions). For the not-so-good portions of the manuscript, many times it was hard to understand the relationship between sentences, and the flow was bad. There were also many sentences that felt like they were responses to the previous reviewers. These could have been more subtly inserted in the manuscript, at times they felt aggressive, making the reading of the manuscript unpleasant. I understand that different have different styles and I understand that multiple rounds of revisions can be frustrating, but I do believe that once the manuscript is written better, it will make a very interesting story important for the fields of neural tube development, NTD pathogenesis and Pol II biology. Please see some comments below.

Major concern

Page 23, line 52: "This finding suggests that for a subset of genes, a larger Pol II pool promotes their transcription." And Page 24, line 11: "On the contrary, a subset of genes with increased Pol II peak density is accompanied by increased transcription in the KO cells, suggesting that the larger Pol II pool tends to augment the transcription of these genes." Page 29, line 33: "The enlarged Pol II pool only selectively influenced the transcription of 106 genes in NPCs, some of which, such as Folh1, are known to be involved in processes critical for neural tube development" I have a hard time believing that the larger Pol II pool is at the origin of the increase Pol II occupancy and differential gene expression. I think that these changes are just due to pathways being activated like P53 would explain Cdkn1a and Gadd45b changes. I know that the authors later in the discussion evoke this possibility, but overall, this claim is too strong. Increased Pol II pool probably has consequences that the authors have not yet discovered, but I don't think it is linked to the transcriptional changes observed. I think that the transcriptomic analyses are "negative data" in a way, and the authors should tone down their interpretation of those results. This does not make this data not interesting, on the contrary, I find it fascinating that such a large increase in the Pol II pool does not have more consequences on transcription.

Minor concerns

1) Page 3 line 36: "shape changes" does not sound very scientific, "morphological changes" or "morphological transformations" might be more appropriate
2) Page 3 line 48: "Mutations/deletions in more than 200 genes are known to cause NTD in mice, although only a limited number of them are associated with human NTD risks according to human genetic studies". This is a strong claim that is not supported by references, it would be helpful to have at least a reference to a review article here.
3) Page 6 line 15: "BRCA1 is reportedly to ubiquitinate POLR2H". "Reportedly" does not sound right here, I believe it should be "reported"
4) Page 8 line 25: "It is customary to include kinky or curly tails as a form of NTD". "customary" does not sound like a good scientific term. Maybe rephrase to "We included kinky and curly tails as forms of NTD {REFS}", or something of the sort.
5) Page 9, line 42: "Randomly testing all KO embryos would include many that had no NTD and would make the data uninterpretable. Only those with exencephaly were the ones who surely had NTD." This is a strong and almost aggressive sentence. The authors should be more subtle in how they write this.
6) Data in figure 4, panels C, D, and G should be analyzed via ANOVAs, repeated student t-tests seem inappropriate.
7) Page 10, line 45: "Cells in the neural tubes cannot be obtained in sufficient number and homogeneity for biochemical analysis". Again, this is a strong claim, probably in response to a reviewer, which in essence is not true. It is possible to obtain homogeneous populations of such cells, it would take considerable time, resources, and effort to do so, but it is not impossible. I don't think it would be needed here, and I agree that neural progenitor cells are a good proxy for the experiments at hand. I think it would be sufficient if the authors removed this sentence and modified the next sentence.
8) Page 10, line 60: "The brain origin of NPCs and the harvesting date are immaterial for the purpose of our study, because these cells need to be expanded in vitro for additional 10-12 days anyways." Another aggressive, strong claim which I think is not needed in the final version of the manuscript.
9) Page 11, line 35: "A set of representative histograms showing the slow cell cycle progression of the KO NPCs are shown at the left. Results from 6 independent experiments are summarized in line graphs at the right." I don't think these two sentences belong to the core text, maybe in the figures?
10) Page 11, line 50: "NPCs will undergo apoptosis upon sudden EGF withdrawal. This is a convenient model to assess NPC apoptosis." Could the authors provide a reference for this?
11) Page 14, line 54: "This makes the increase of POLR2A protein levels more prominent." I am not sure of what the authors mean by this sentence. Please rephrase.
12) Figure 7, panel A: The "Cnot1" gene is on the heatmap twice. I am not sure what happened here.
13) Page 20, line 56: "gene ontology", not "gene ontogeny"
14) Page 36, line 10: "This makes the phenotype comparison between mice and humans impossible and not sensible." Another example of a strong claim in response to reviewers, which I think should be introduced more subtly. In essence I agree with the authors, but it should be written better. Actually, I think this whole paragraph should be written differently, in a less definitive and defensive tone.

**Authors’ response**

*Reviewer #1: To me, overall, the data is strong but the writing of this manuscript has two facets, it can be either very good (the biochemistry and human data part) or on the contrary really hard to read (the mouse phenotyping portions). For the not-so-good portions of the manuscript, many times it was hard to understand the relationship between sentences, and the flow was bad. There were also many sentences that felt like they were responses to the previous reviewers. These could have been more subtly inserted in the manuscript, at times they felt aggressive, making the reading of the manuscript unpleasant. I understand that different have different styles and I understand that multiple rounds of revisions can be frustrating, but I do believe that once the manuscript is written better, it will make a very interesting story important for the fields of neural tube development, NTD pathogenesis and Pol II biology. Please see some comments below.*

Indeed, some texts, which at times seem abrupt and defensive, are inserted in response to previous reviewers' comments. We have now adopted a neutral tone and smoothed the flow of the narrative. The mouse phenotype part involves some rather complicated calculations for the NTD incidence rates in the KO mice, at weaning and on e13.5, based on live and dead mice. The calculations might have caused some confusion. We have now improved the writing and tried to make it easier to comprehend.

*Major concern*

*Page 23, line 52: "This finding suggests that for a subset of genes, a larger Pol II pool promotes their transcription." And Page 24, line 11: "On the contrary, a subset of genes with increased Pol II peak density is accompanied by increased transcription in the KO cells, suggesting that the larger Pol II pool tends to augment the transcription of these genes." Page 29, line 33: "The enlarged Pol II pool only selectively influenced the transcription of 106 genes in NPCs, some of which, such as Folh1, are known to be involved in processes critical for neural tube development" I have a hard time believing that the larger Pol II pool is at the origin of the increase Pol II occupancy and differential gene expression. I think that these changes are just due to pathways being activated like P53 would explain Cdkn1a and Gadd45b changes. I know that the authors later in the discussion evoke this possibility, but overall, this claim is too strong. Increased Pol II pool probably has consequences that the authors have not yet discovered, but I don't think it is linked to the transcriptional changes observed. I think that the transcriptomic analyses are "negative data" in a way, and the authors should tone down their interpretation of those results. This does not make this data not interesting, on the contrary, I find it fascinating that such a large increase in the Pol II pool does not have more consequences on transcription.*

We agree with the reviewer that, at this time, we do not have enough evidence to attribute the cause of NTD only to the larger Pol II pool. It is totally possible that the E3 has other targets, and the NTD phenotype is the sum of its direct and indirect effects on various target molecules, not only NPCs but also other tissues or organs at different times of NTD development. We have moderated or eliminated this claim in the places pointed out by the reviewer.

*Minor concerns*

*1) Page 3 line 36: "shape changes" does not sound very scientific, "morphological changes" or "morphological transformations" might be more appropriate.*

Revised as suggested.

*2) Page 3 line 48: "Mutations/deletions in more than 200 genes are known to cause NTD in mice, although only a limited number of them are associated with human NTD risks according to human genetic studies". This is a strong claim that is not supported by references, it would be helpful to have at least a reference to a review article here.*

Two references were added.

*3) Page 6 line 15: "BRCA1 is reportedly to ubiquitinate POLR2H". "Reportedly" does not sound right here, I believe it should be "reported"*

Revised as suggested.

*4) Page 8 line 25: "It is customary to include kinky or curly tails as a form of NTD". "customary" does not sound like a good scientific term. Maybe rephrase to "We included kinky and curly tails as forms of NTD {REFS}", or something of the sort.*

Rephrased.

*5) Page 9, line 42: "Randomly testing all KO embryos would include many that had no NTD and would make the data uninterpretable. Only those with exencephaly were the ones who surely had NTD." This is a strong and almost aggressive sentence. The authors should be more subtle in how they write this.*

The sentence has been removed, as it is not really necessary. It was added to address one reviewer's request to test all the KO embryos. I think most people will not ask for such an experiment.

*6) Data in figure 4, panels C, D, and G should be analyzed via ANOVAs, repeated student t-tests seem inappropriate.*

The reviewer probably meant panels in Figure 2. Two-factor with replication ANOVA has been employed now for statistical analysis in Figs. 2C, 2D and 2D.

*7) Page 10, line 45: "Cells in the neural tubes cannot be obtained in sufficient number and homogeneity for biochemical analysis". Again, this is a strong claim, probably in response to a reviewer, which in essence is not true. It is possible to obtain homogeneous populations of such cells, it would take considerable time, resources, and effort to do so, but it is not impossible. I don't think it would be needed here, and I agree that neural progenitor cells are a good proxy for the experiments at hand. I think it would be sufficient if the authors removed this sentence and modified the next sentence.*

Yes, this was a response to a reviewer's comment. This sentence has been rephrased as follows.

"It is very challenging to obtain neural tube cells in sufficient numbers and homogeneity for biochemical analysis."

*8) Page 10, line 60: "The brain origin of NPCs and the harvesting date are immaterial for the purpose of our study, because these cells need to be expanded in vitro for additional 10-12 days anyways." Another aggressive, strong claim which I think is not needed in the final version of the manuscript.*

Again, this was a response to a reviewer. It has been deleted in the final version.

*9) Page 11, line 35: "A set of representative histograms showing the slow cell cycle progression of the KO NPCs are shown at the left. Results from 6 independent experiments are summarized in line graphs at the right." I don't think these two sentences belong to the core text, maybe in the figures?*

Yes, these sentences belong to the figure legend. They were in the result section by mistake and have been removed.

*10) Page 11, line 50: "NPCs will undergo apoptosis upon sudden EGF withdrawal. This is a convenient model to assess NPC apoptosis." Could the authors provide a reference for this?*

References provided.

*11) Page 14, line 54: "This makes the increase of POLR2A protein levels more prominent." I am not sure of what the authors mean by this sentence. Please rephrase.*

This sentence has been rephrased.

*12) Figure 7, panel A: The "Cnot1" gene is on the heatmap twice. I am not sure what happened here.*

Our RNA-seq reads were aligned at the transcript level, and more than 40,000 transcripts were obtained. Many genes have more than one transcript mapped. In the case of Cnot1, it has a transcript that is significantly upregulated and another one (at a different part of the gene) that is downregulated. Therefore, it appeared twice in the heatmap, one in the upregulated category (red) and one in the downregulated category (blue). This probably reflects the dysregulation of two different isoforms of this gene. This is now explained in the Results section.

*13) Page 20, line 56: "gene ontology", not "gene ontogeny"*

This typo has been corrected.

*14) Page 36, line 10: "This makes the phenotype comparison between mice and humans impossible and not sensible." Another example of a strong claim in response to reviewers, which I think should be introduced more subtly. In essence I agree with the authors, but it should be written better. Actually, I think this whole paragraph should be written differently, in a less definitive and defensive tone.*

Yes, this was added in response to a reviewer's question about why the human and mouse phenotypes are different. We were a little frustrated by such a question. We have now rewritten the paragraph and moderated the narrative.
